# New ion radii for oxides and oxysalts, fluorides, chlorides and nitrides

**DOI:** 10.1107/S2052520624005080

**Published:** 2024-07-15

**Authors:** Frank C. Hawthorne, Olivier C. Gagné

**Affiliations:** a Earth Sciences University of Manitoba 125 Dysart Road Winnipeg ManitobaR3T 2N2 Canada; Georgetown University, USA

**Keywords:** electron density, radius ratio, experimental ion radii, theoretical ion radii

## Abstract

Ion radii are derived here from the characteristic (grand mean) bond lengths for (i) 135 ions bonded to oxygen in 459 configurations (on the basis of coordination number) using 177 143 bond lengths extracted from 30 805 ordered coordination polyhedra from 9210 crystal structures; and (ii) 76 ions bonded to nitro­gen in 137 configurations using 4048 bond lengths extracted from 875 ordered coordination polyhedra from 434 crystal structures. There are two broad categories of use for ion radii: (1) those methods which use the relative sizes of cation and anion radii to predict local atomic arrangements; (2) those methods which compare the radii of different cations (or the radii of different anions) to predict local atomic arrangements. There is much uncertainty with regard to the relative sizes of cations and anions, giving rise to the common failure of type (1) methods, *e.g*. Pauling’s first rule which purports to relate the coordination adopted by cations to the radius ratio of the constituent cation and anion. Conversely, type (2) methods, which involve comparing the sizes of different cations with each other (or different anions with each other), can give very accurate predictions of site occupancies, physical properties *etc*. Methods belonging to type (2) can equally well use the characteristic bond lengths themselves (from which the radii are derived) in place of radii to develop correlations and predict crystal properties. Extensive quantum-mechanical calculations of electron density in crystals in the literature indicate that the radii of both cations and anions are quite variable with local arrangement, suggesting significant problems with any use of ion radii. However, the dichotomy between the experimentally derived ion radii and the quantum-mechanical calculations of electron density in crystals is removed by the recognition that ion radii are pr­oxy variables for characteristic bond lengths in type (2) relations.

## Abstract 2

Ion radii are derived from observed mean bond lengths (*characteristic bond lengths*) for 135 ions bonded to oxygen, fluorine, hydroxyl, chlorine and nitro­gen. Radii derived from quantum-mechanical calculations do not agree with radii derived from experimentally determined bond lengths. However, this problem is removed by recognition that ion radii determined from characteristic bond lengths are not a measure of the sizes of ions but proxy variables for characteristic bond lengths.

## 1. Introduction

With the advent of X-ray diffraction, the distances between atoms in crystal structures gave a sense of the sizes of these atoms. However, assuming spherical atoms and simple chemical compositions, the radius of one atom must be specified in order to derive the radius of the atom to which it is bonded. Much ingenuity has been expended on deriving radii for simple anions, particularly O^2−^. However, there has been a lack of unanimity concerning methods of assigning such radii, giving rise to different sets of both cation and anion radii, a situation that has continued to the present day. At the same time, quantum-mechanical methods were developed, giving insight into the detailed behaviour of electron density and enabling certain aspects of atom size (among many other topics) to be addressed. There are strong parallels between these two approaches, for example the strong correlation between the calculated electron density accumulated at the bond-critical point (see below) and the Pauling bond-strength of that bond. However, there are also differences from which uncertainties arise concerning the definitions of *chemical bond* and *ion radius*. Here we address the issue of ion radius from both perspectives and present a comprehensive table of ion radii derived from experimental interatomic distances for the anions O^2−^, (OH)^−^, F^−^, Cl^−^ and N^3−^.

In the interest of clarity, we define certain terms that we use in the following text:

*Coordination number*: The number of counterions bonded to an ion.

*Coordination polyhedron*: The arrangement of counterions around an ion. The coordination number of an ion is indicated by the number of counterions, *n*, enclosed in square brackets and written as a superscripted prefix: e.g. ^[6]^Mg^2+^.

*Ion configuration*: A unique arrangement of ion type and coordination number.

*Valence and oxidation state:* These terms are commonly used synonymously in the literature. IUPAC (2019 ▸) (the International Union of Pure and Applied Chemistry) defines valence as follows: ‘The maximum number of univalent atoms (originally hydrogen or chlorine atoms) that may combine with an atom of the element under consideration, or with a fragment, or for which an atom of this element can be substituted’. Two other accepted definitions are: ‘…the number of hydrogen atoms that can combine with an element in a binary hydride or twice the number of oxygen atoms combining with an element in its oxide or oxides’ (Greenwood & Earnshaw, 1997 ▸, p. 27), and ‘…the number of electrons that an atom uses in bonding’ (Parkin, 2006 ▸, p. 791). IUPAC (2019 ▸) defines oxidation state as follows: ‘the charge on an atom after ionic approximation of its heteronuclear bonds’. Whichever definition one accepts for valence, it is clear that valence does not have a sign whereas oxidation state (number) does have a sign (or is 0). Here we will use the term oxidation state.

## 2. The lengths of chemical bonds

It has long been assumed that nearest-neighbour atoms in a crystal are linked together by chemical bonds. IUPAC (2019 ▸) gives the following as a ‘definition’ of a chemical bond: *When forces acting between two atoms or groups of atoms lead to the formation of a stable independent molecular entity, a chemical bond is considered to exist between these atoms or groups*. This is a statement, not a definition. A definition needs to have the following grammatical structure: ‘A chemical bond is …’. Moreover, there is a problem with this definition/statement: The coordination of Na by six Cl in the crystal structure of halite is not ‘a stable independent molecular entity’, it is part of an extended array of bonded atoms that forms a crystal.

When discussing interatomic distances, Gagné & Hawthorne (2016 ▸, p. 603) made the following statement: ‘There is no rigorous definition of a chemical bond that is useful in the context of the present work which deals with some hundreds of thousands of observed interatomic distances. The decision on whether or not a specific interatomic distance corresponds to a chemical bond is made in terms of the local environment of the constituent atoms, *e.g.* is the distance consistent with a specific coordination number of the central ion, and is the valence-sum rule (Brown, 2016 ▸) reasonably well satisfied for the constituent ions? These are the criteria that are generally used for listing bond lengths in crystal-structure papers.’ The ion radii listed in this paper are derived from bond lengths selected according to these criteria.

## 3. Ions and the ionic-bonding model

There has been much criticism of Pauling’s rules (Pauling, 1929 ▸) during the last 95 years, particularly in terms of their perceived association with the model of ionic bonding. In this model, electrons are transferred in integral numbers from atoms of low electronegativity to atoms of high electronegativity, imparting integral charges to the constituent atoms which are held together by electrostatic forces between the resultant ions. This is the ionic model in which the bonding is considered as 100% ionic. An alternative view (Albright *et al.*, 2013 ▸) is that the valence electron-density is not transferred from one bonded atom to another but is used to form molecular orbitals that bond the atoms together, and the bonds are considered as covalent. The problem with both these approaches at the elementary level is that they consider electrons in bonded systems as integral entities and charge redistribution is described in terms of integral charges. In contrast with this picture, charge-density refinement of inorganic crystal structures (*e.g*. Table 1 ▸ for pyroxenes) shows that charges on the atoms are non-integral in accord with the view that these structures are neither ‘ionic’ nor ‘covalent’.

In an isolated neutral atom, the electrons occur in atomic orbitals and are held in the atom by the electrostatic interaction between the electron density and the protons of the nucleus. When two neutral atoms approach each other closely, the outer (valence) electron-density of one atom is also attracted to the protons of the second atom and is partly delocalized, hybridizing to form molecular orbitals (MO model) or sharing electron density between the two atoms to form a chemical bond (bond-valence model). Thus in both models, valence electron-density is shared between the bonded atoms. The term ‘ionic radius’ is not suitable as a descriptor of the sizes of these two atoms as it associates the size and charge of the atom with the ionic model, whereas this is not the case. We will use the term ‘ionic radius’ to refer to radii that predate the present work, and the term ‘ion radius’ to the radii of the present work in order to distance the radius of an ion derived here from the ionic model. Furthermore, we will use the term ‘empirical ionic radius’ to refer specifically to the values of Shannon (1976 ▸).

## 4. The sizes of atoms

**Figure 1:**
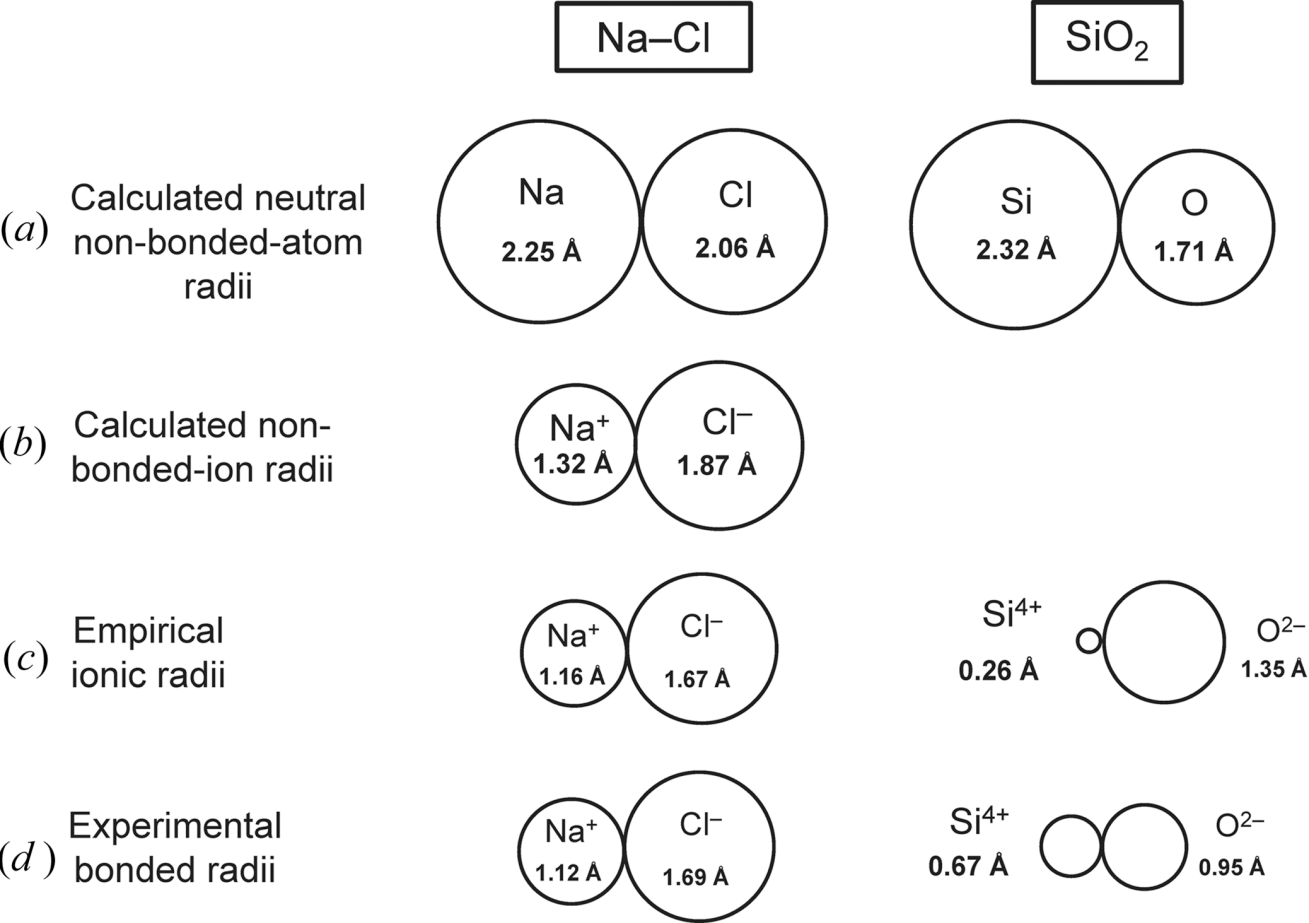
Comparison of the sizes (radii) of atoms: (*a*) calculated neutral non-bonded-atom radii, (*b*) calculated non-bonded-ion radii, (*c*) empirical ionic radii and (*d*) experimental bonded radii. Values for (*a*) and (*b*) are taken from Rahm *et al.* (2017 ▸), values for (*c*) from Shannon (1976 ▸), values for (*d*) from Gibbs *et al.* (2013 ▸).

The size of a specific atom depends on (i) its oxidation state, (ii) whether it is bonded to other atoms, or whether it is non-bonded (*i.e.* isolated), and (iii) if bonded, several other factors involving its electronic structure and environment. In order to give a sense of the relative magnitudes of these factors, Fig. 1 ▸ shows a series of different radii for Na, Cl, Si and O. The calculated non-bonded radii are taken from Rahm *et al.* (2017 ▸); note that adjacent Na^0^ and Cl^0^ are drawn touching each other in order to more easily gauge differences in radii; they are not bonded [Fig. 1 ▸(*a*)]. The radii of the non-bonded ionized atoms [Fig. 1 ▸(*b*)] are far smaller than those of the neutral atoms, indicating the diffuse nature of the valence electron-density in both Na^0^ and Cl^0^. The empirical ionic radii of Shannon (1976 ▸) for ^[6]^Na^+^ and ^[6]^Cl^−^ [Fig. 1 ▸(*c*)] are smaller than the non-bonded ion radii. The difference between the neutral non-bonded radius for Si^0^ [Fig. 1 ▸(*a*)] and the empirical ionic radius for ^[4]^Si^4+^ [Fig. 1 ▸(*c*)] is extreme (the latter being smaller than the former by a factor of 9). Experimental bonded radii may be derived from the minimum in experimental electron-density between bonded atoms. For Na^+^ and Cl^−^, the experimental bonded-radii [Fig. 1 ▸(*d*)] are only slightly different from the empirical ionic radii [Fig. 1 ▸(*c*)], whereas for ^[4]^Si^4+^ and O^2−^, the experimental bonded-radii [Fig. 1 ▸(*d*)] are significantly different from the corresponding empirical ionic radii [Fig. 1 ▸(*c*)] by a factor of 2.6 for ^[4]^Si^4+^. Furthermore, the length of the bond pair for Si^4+^–O^2−^, *e.g.* (0.26 + 1.35) × 2 = 3.24 Å, is less than the diameter of Si^0^, 2.32 × 2 = 4.64 Å, emphasizing the diffuse nature of the valence electron-density in isolated neutral atoms.

### 4.1. Ionic radii and empirical ionic radii

Bragg (1920 ▸) found that interatomic distances in crystals can be reproduced by the sum of the radii of the bonded atoms. In addition, he derived a set of ionic radii where the sum of the radii reproduced the bond lengths for many crystals to within ∼0.06 Å. Landé (1920 ▸) assumed that halogen ions are in mutual contact in the structures of the lithium halogenides and assigned the sizes of ions accordingly. Hüttig (1920 ▸) concluded that the co­ordination number adopted by cations is determined by radius-ratio considerations; the larger the ratio, the larger the expected coordination number of the cations. Using the connection be­tween mole refraction and ionic volume, Wasastjerna (1923 ▸) produced a more extensive set of ionic radii that was extended by Goldschmidt (1926 ▸) and Pauling (1927 ▸). Collectively, this work concluded that anions are larger (over 1.35 Å) than cations. Goldschmidt (1926 ▸) used Hüttig’s (1920 ▸) coordination-number arguments to predict coordination numbers for a wide range of cations. Pauling (1929 ▸) collected these ideas, developed others, and consolidated them as a set of relatively simple yet powerful rules for understanding and predicting stable atomic arrange­ments in oxide-based minerals. The ionic radii of the metal ions were assumed to decrease systematically from left to right in each row of the Periodic Table and to increase as the row numbers of the atoms increase. Pauling (1929 ▸) also noted that indi­vidual metal–oxygen (*M*–O) bond lengths tend to decrease with increasing oxidation state and decreasing coordination number of the *M* cation. Ahrens (1952 ▸) produced the next comprehensive set of ionic radii using a combination of experimental interatomic distances and interpolation/extrapolation involving correlations of ionic radii with ionization potentials of the constituent ions. These radii were widely used until Shannon & Prewitt (1969 ▸) and Shannon (1976 ▸) produced the widely used set of empirical ionic radii using experimental interatomic distances, interpolation/extrapolation involving correlations of radii with cell volumes, coordination number and oxidation state, and values from Ahrens (1952 ▸).

In most of the approaches described above, most ionic radii were derived by subtracting a radius for O^2−^ from observed interatomic distances. Various experimentally based values had been used for the radius of O^2−^ since ionic radii were first derived, but the compilation by Shannon (1976 ▸) used radii for O^2−^ that are dependent on the coordination number of O^2−^: [2] = 1.35 < *r*O^2−^ < [6] = 1.42 Å. Next, we will review the available evidence for coordination-dependent radii for O^2−^.

#### 4.1.1. The empirical radius of O^2−^

Following the suggestion of Smith & Bailey (1963 ▸) that variations in mean bond length of Si^4+^O_4_ and Al^3+^O_4_ tetrahedra in feldspars are related to the degree of polymerization of the constituent tetrahedra, Shannon & Prewitt (1969 ▸) showed 〈Si^4+^–O^2−^〉 as a linear function of the coordination of O^2−^ (for four data points), and developed coordination-dependent ionic radii for O^2−^. Brown & Gibbs (1969 ▸) developed a correlation between mean bond length and the mean anion-coordination number of the Si^4+^O_4_ tetrahedra in 46 silicate structures, omitting several Na-silicates. Shannon & Prewitt (1969 ▸) used this correlation to justify using radii for O^2−^ that vary as a function of coordination number in developing their set of empirical ionic radii, and Shannon (1976 ▸) listed radii for O^2−^ from ^[2]^1.35 Å to ^[8]^1.42 Å.

Gagné & Hawthorne (2017*b* ▸) examined the variation in mean bond length as a function of (i) anion-coordination number, (ii) the electronegativity of the nearest-neighbour cations, (iii) bond-length distortion, (iv) the ionization energy of the nearest-neighbour cations, and (v) the differences in bond topology, for 55 ion configurations. They also examined the effect of sample size on the statistical significance of the results as measured by the *p*-value for the null hypothesis that the slope of the correlation between variables is equal to zero, and by the value of *R*^2^ which is a measure of the fraction of variation of the dependent parameter that can be attributed to the independent variable.

**Figure 2:**
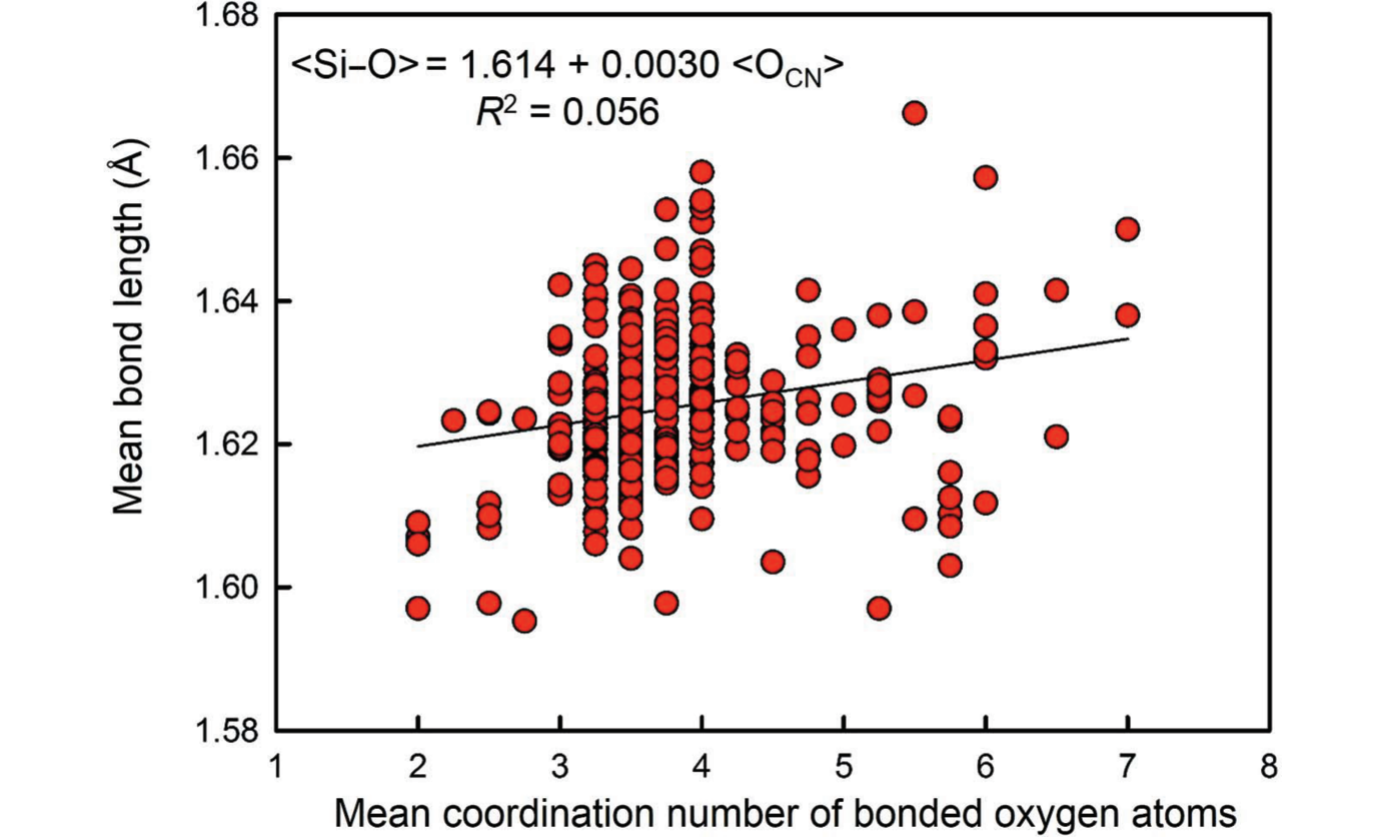
Mean ^[4]^Si–O distance versus mean coordination number of the bonded oxygen atoms for 334 SiO_4_ coordination polyhedra; after Gagné & Hawthorne (2017*b* ▸).

**Figure 3:**
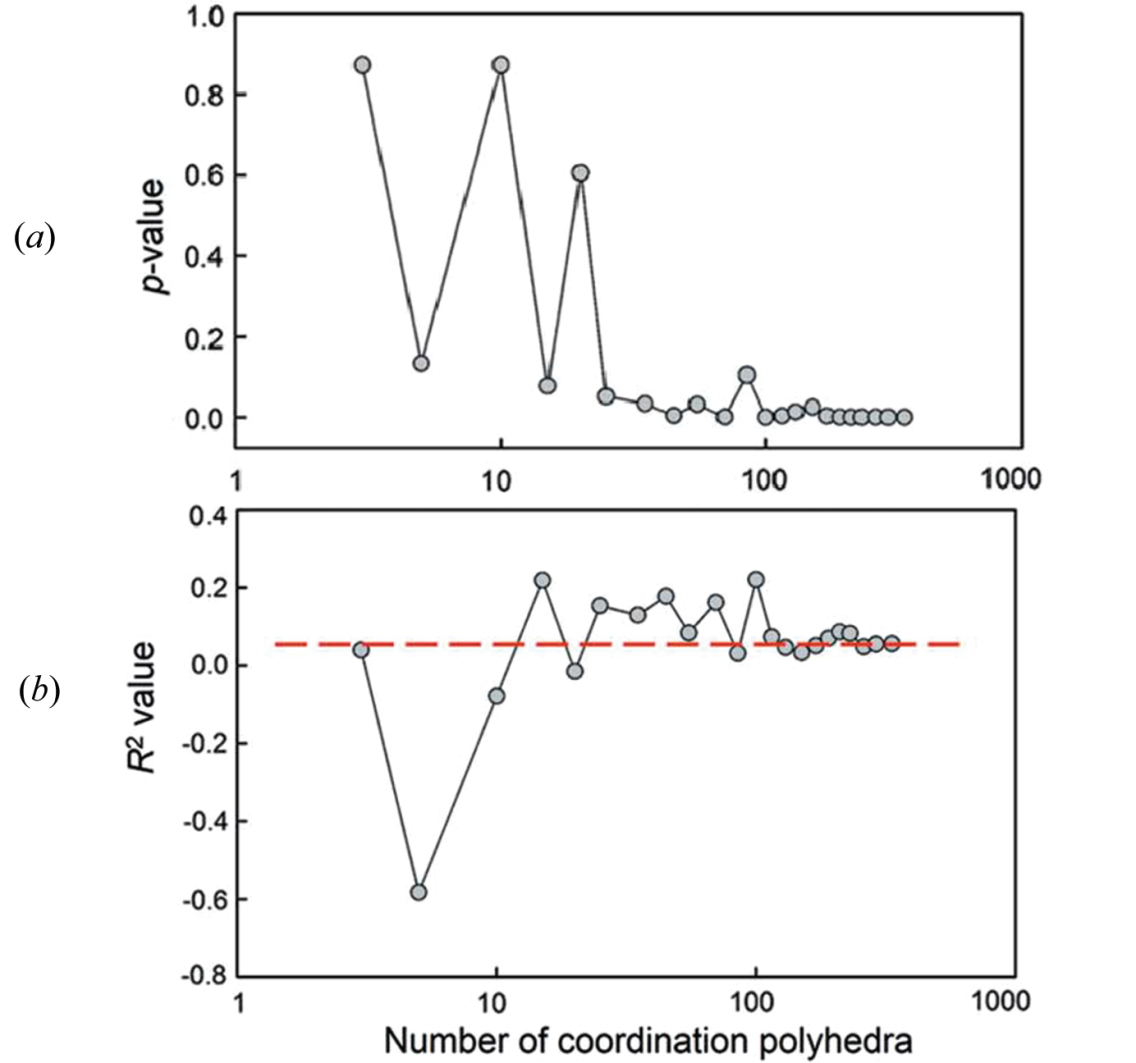
Effect of sample size on the statistical significance of the correlations between mean ^[4]^Si–O and mean coordination number of the constituent O atoms: as measured (*a*) by *p*-values and (*b*) by *R*^2^ values, where the dashed line shows the value for the parent distribution (*n* = 334); after Gagné & Hawthorne (2017*b* ▸).

**Figure 4:**
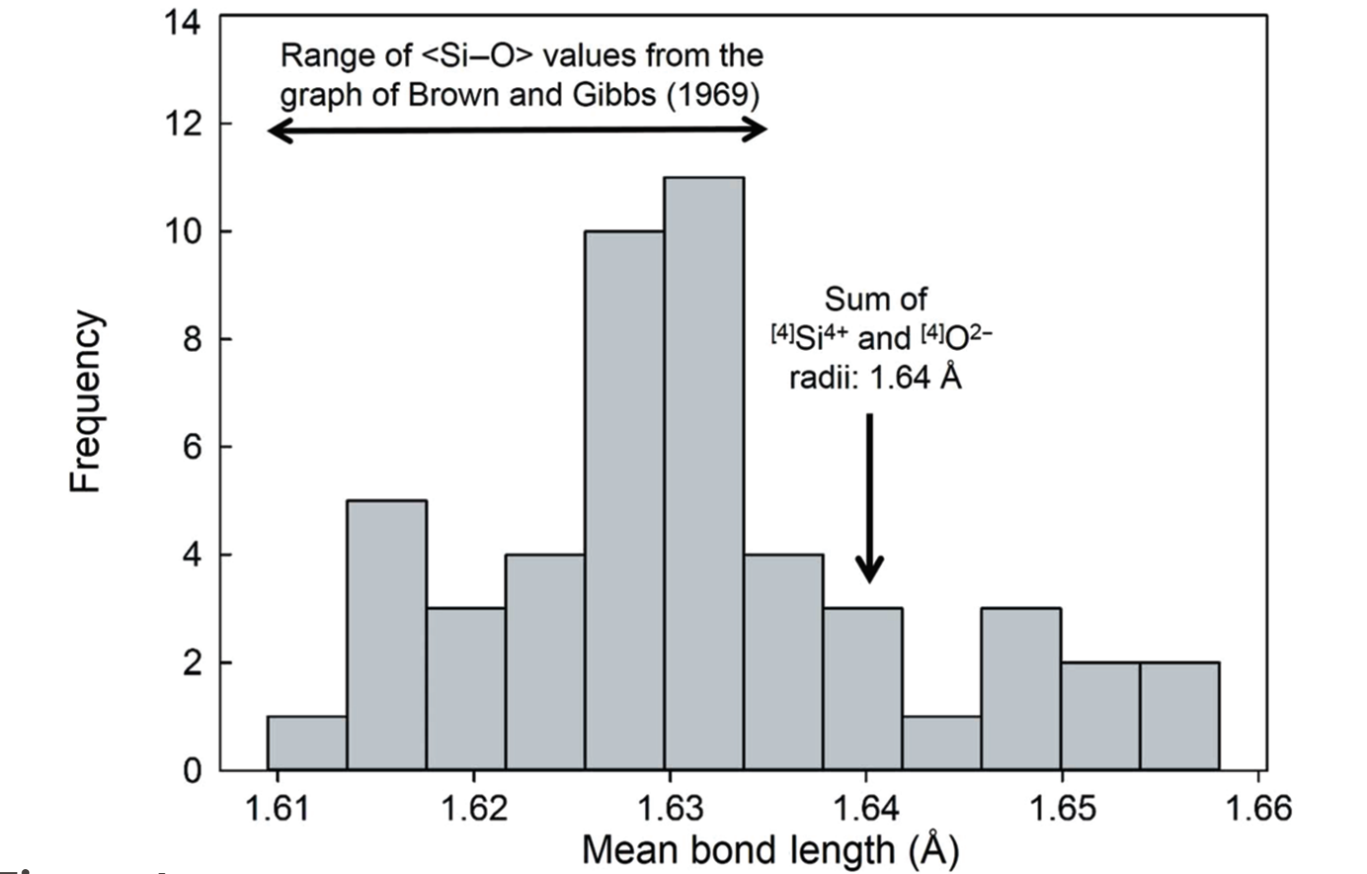
Distribution of mean ^[4]^Si–O distances for structures with a mean coordination number for O^2−^ of [4]. The range of mean Si–O values taken from the trend line on the graph of Brown & Gibbs (1969 ▸), and the sum of the ^[4]^Si^4+^ and ^[4]^O^2−^ radii from Shannon (1976 ▸) are shown; from Gagné & Hawthorne (2017*b* ▸).

Fig. 2 ▸ shows the variation in 〈Si^4+^–O^2−^〉 (= mean Si^4+^–O^2−^ distance) distances as a function of the constituent-anion coordination number for 334 tetrahedra, with the data for the regression shown. From this data, we may evaluate the effect of sample size on the statistics of the fitting process. We ran the regression for a series of different sample sizes and the results are shown in Fig. 3 ▸. The *p*-value fluctuates wildly at small sample sizes [Fig. 3 ▸(*a*)] and only settles down to a constant value (that is equal to the value for the complete data set) for sample sizes > 100. The *R*^2^ value shows similar behaviour and converges on the *R*^2^ value for the complete data set [shown by the dashed red line in Fig. 3 ▸(*b*)] for sample sizes > 100. Both these statistical measures indicate that spurious correlations may arise for smaller sample sets, particularly if the distribution of bond lengths in the complete data set is multimodal. These results indicate that previous results on the effect of anion coordination on 〈Si^4+^–O^2−^〉 distances are not dependable as they were obtained on sample sets that are too small to have reliable statistics. This finding is emphasized in Fig. 4 ▸ which shows the distribution of 49 〈Si^4+^–O^2−^〉 distances with an O^2−^ coordination of [4]. The range is twice that of the data given by Brown & Gibbs (1969 ▸) for which [2] ≤ O^2−^ ≤ [4]. Moreover, the sum of the Shannon (1976 ▸) radii, 0.26 + 1.38 = 1.64 Å, is outside the range of values given by Brown & Gibbs (1969 ▸) and does not correspond with the average value (1.631 Å) of the data in Fig. 4 ▸.

So what do we conclude from the above discussion? We conclude that any effect of variation in constituent-anion coordination number on variation in 〈Si^4+^–O^2−^〉 distances is minor compared with other stereochemical effects (particularly differences in local bond topology, Gagné & Hawthorne, 2020 ▸) and is not apparent in the data presently available. Moreover, the results of Fig. 3 ▸ indicate that these conclusions are unlikely to be changed by additional data. Thus we conclude that a single value for the radius of O^2−^ is effective for SiO_4_ tetrahedra and, by extension, for the other ions bonded to O^2−^.

### 4.2. Bonded radii

**Figure 5:**
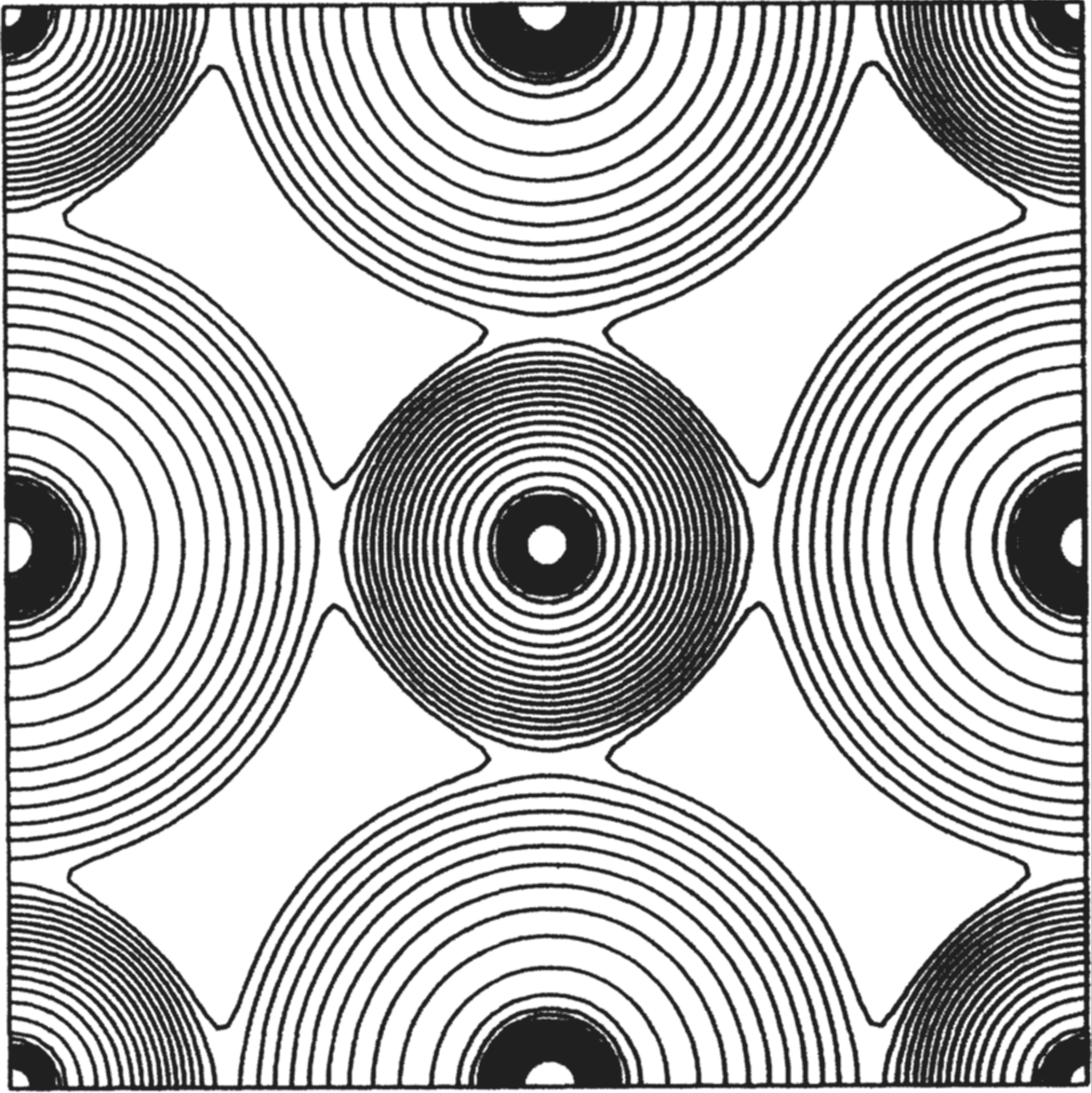
Electron density in NaCl projected onto (100), modified from Jansen & Freeman (1986 ▸).

In a high-symmetry structure in which all bonds within a holosymmetric polyhedron are of equal length, the radii of the constituent ions can be read from a map of the distribution of electron density. Thus in NaCl (Fig. 5 ▸), the ions are assumed to be spherical and the minimum in electron density along the Na^+^–Cl^−^ bond defines the radii of ^[6]^Na^+^ and ^[6]^Cl^−^. However, in anisodesmic structures in which the bonds are of very different strength, this will not be the case. Pauling & Hendricks (1925 ▸) argued that in the structures of corundum and its isotypes, the occurrence of symmetrically distinct bonds in the coordination polyhedra of the cations should result in the radii of the cations being different along bonds of different length. There is no intrinsic reason why such an effect should be limited to cations, and if the argument is accepted, one would also expect the radii of anions to be different along bonds of different length.

#### 4.2.1. Theoretical bonded radii

The electron density in a crystal can be calculated by imposing periodicity on its wavefunctions (as Bloch functions) in a quantum-mechanical calculation. The calculated electron density is a quantum-mechanical observable and examination of such electron-density distributions shows a series of stationary points at which the electron density is at a minimum with respect to some directions and at a maximum with respect to other directions, *i.e.* they are *saddle points* (Runtz *et al.*, 1977 ▸). Saddle points normally occur on or near lines joining the nuclei of pairs of atoms that are (thought to be) bonded to each other. Any line of steepest descent that terminates at a saddle point is defined as a *gradient path*. The two gradient paths which originate at the same saddle point and end at each of two nuclei define a *bond path*, and the included saddle point is called a *bond critical point* (Bader, 2009 ▸). Note that the bond critical point will not, in general, lie on the internuclear axis unless constrained to do so by symmetry, and thus the bond path joining the two nuclei will deviate from that internuclear axis (Runtz *et al.*, 1977 ▸). It is important to distinguish between a chemical bond, the definition of which is fraught with complications, and a bond path which is a quantum-mechanical observable. According to Bader (2009 ▸), a bond path is not a chemical bond, it is an indicator of chemical bonding (note the analogy with the rather oblique IUPAC ‘definition’ of a chemical bond discussed above).

**Figure 6:**
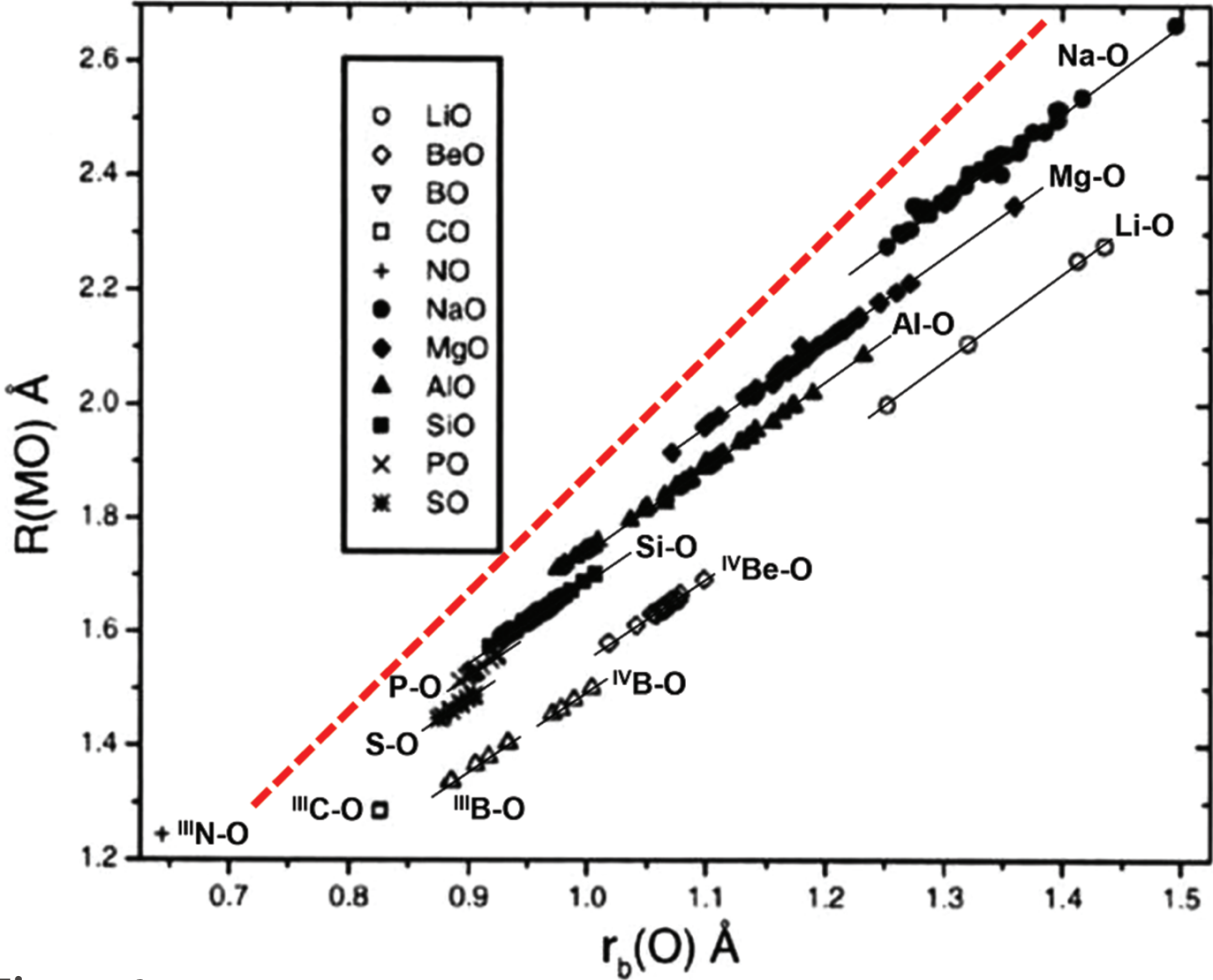
Experimental *M*–O bond lengths, *R*(*M*–O) Å, versus the bonded radius, *r*_b_(O), of O atoms bonded to second row (Li, Be, B,…) and third row (Na, Mg, Al,…) cations for silicate and oxide structures (after Gibbs *et al.*, 2001 ▸). Copyright (2001) Mineralogical Society of America.

**Figure 7:**
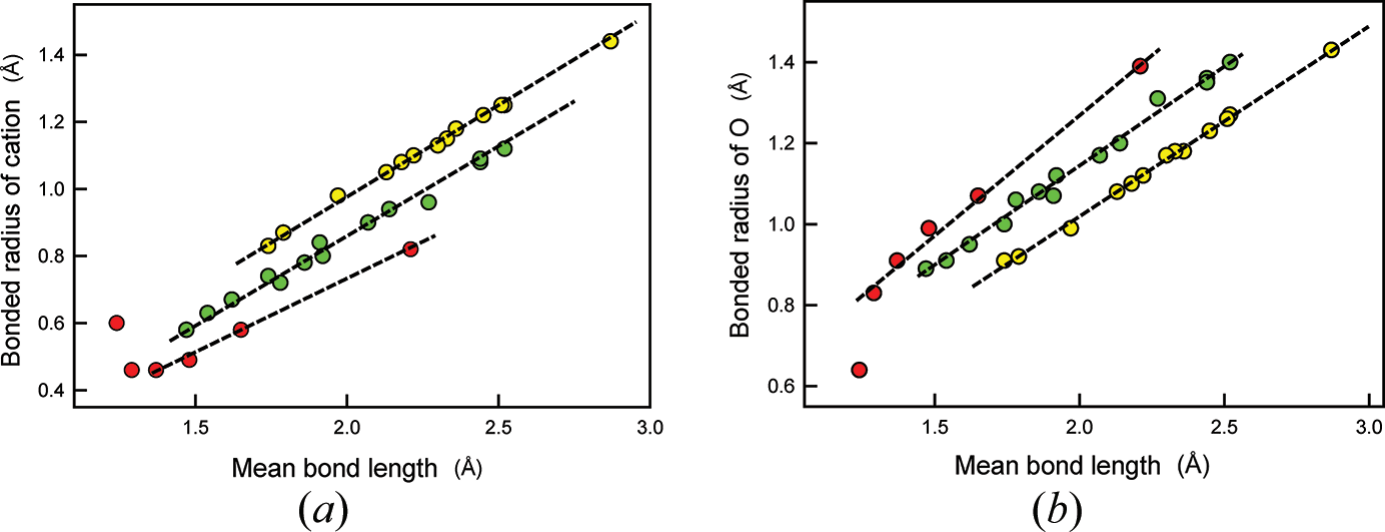
(*a*) Variation in calculated bonded radii for second- (red), third- (green) and fourth- (yellow) row cations bonded to O^2−^ as a function of experimental 〈*M*–O〉 bond lengths; (*b*) variation in calculated bonded radii for O^2−^ bonded to second- (red), third- (green) and fourth- (yellow) row cations; data for silicate and oxide structures (modified from Gibbs *et al.*, 2013 ▸).

The variation of bonded radii in anisodesmic structures has been investigated extensively by Gibbs and co-workers (*e.g.* Gibbs *et al.*, 2001 ▸, 2013 ▸, 2014 ▸) who showed that (i) the calculated bonded radii of both individual cations and anions are not fixed but vary with interatomic distance, and (ii) the calculated bonded radius of O is linearly related to the associated bond length for individual cations of the second, third and fourth rows of the Periodic Table (Fig. 6 ▸). The dashed red line in Fig. 6 ▸ is drawn parallel to the mean trends for the elements of the third and fourth rows of the Periodic Table. As shown by Gibbs *et al.* (2013 ▸, 2014 ▸), there are analogous relations for the mean calculated bonded radius of O and the experimental mean bond lengths [Figs. 7 ▸(*a*) and 7 ▸(*b*)]. and the trends for the cations of the third and fourth rows of the Periodic Table are parallel to the dashed red line in Fig. 6 ▸.

**Figure 8:**
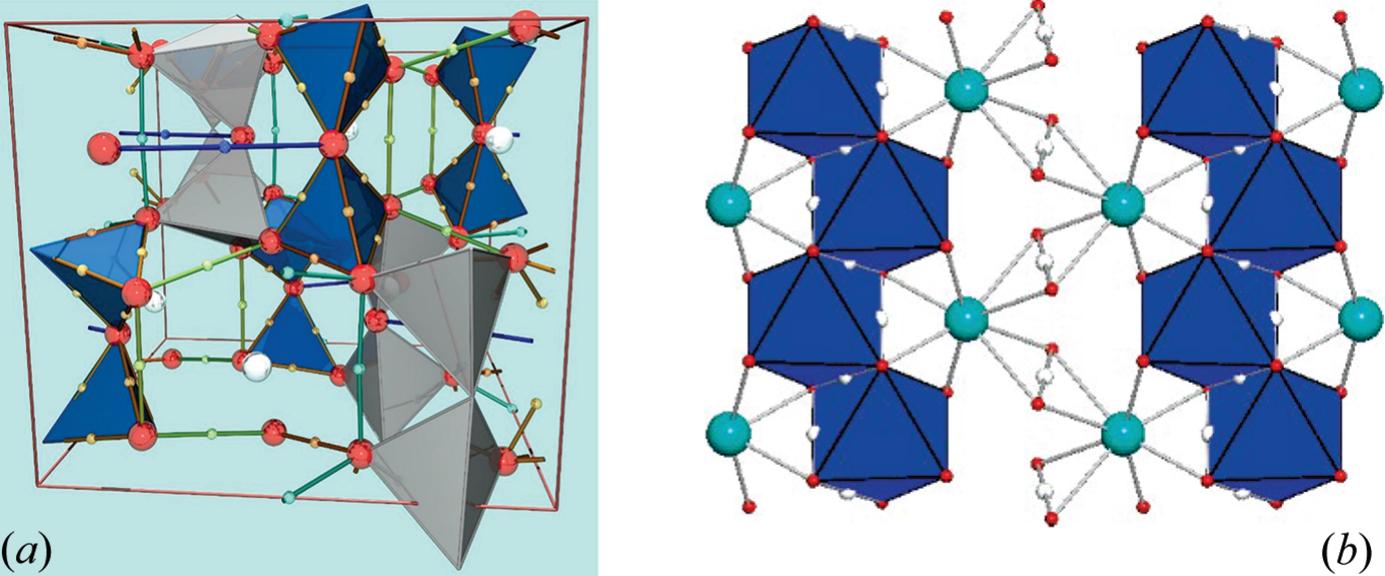
(*a*) The crystal structure of danburite showing the position of the O–O bond critical points (small pale-brown and pale-green spheres) and the O–O bond paths (brown and green lines passing through bond critical points). The dark tetrahedra are the BO_4_ groups, and the light tetrahedra are the SiO_4_ groups. Reprinted with permission from Luaña *et al.* (2003 ▸). Copyright (2003) American Chemical Society. (*b*) The crystal structure of diopside showing the position of the O–O bond critical points (small white spheres) and the O–O bond paths (lines passing through the bond critical point). Reprinted with permission from Gibbs *et al.* (2008 ▸). Copyright (2008) American Chemical Society.

The trend for second-row cations in Fig. 7 ▸(*b*) is drastically different from the trends for the third- and fourth-row cations: (i) the trend for the second-row cations is non-linear; (ii) the general trend for the second-row cations is not parallel to the trends for the third- and fourth-row cations; (iii) the non-linearity for the second-row cations increases with decreasing mean bond length. Calculations for structures with small second-row cations have shown the presence of bond critical points between O–O edges of the oxyanion groups and between other short O–O distances [*e.g.* Figs. 8 ▸(*a*) and 8 ▸(*b*)]. Pakiari & Eskandari (2007 ▸) showed the presence of bond critical points between O–O edges in enol forms of *cis*-β-diketones which result in up to 16 kcal mol^−1^ of local stabilization to the total energy of the molecule, and in which the delocalization index, a measure of the exchange of electrons between the constituent O atoms, is highly correlated with the amount of electron density at the bond critical point. Conversely, Gibbs *et al.* (2000 ▸, 2008 ▸) examined the issue of bond critical points between O–O edges primarily in silicates and stated that ‘The occurrence of O–O bond paths shared in common between equivalent coordination polyhedra suggests that they may be grounded in some cases on factors other than bonded interactions.’ (Gibbs *et al.* 2008 ▸, p. 3693).

**Figure 9:**
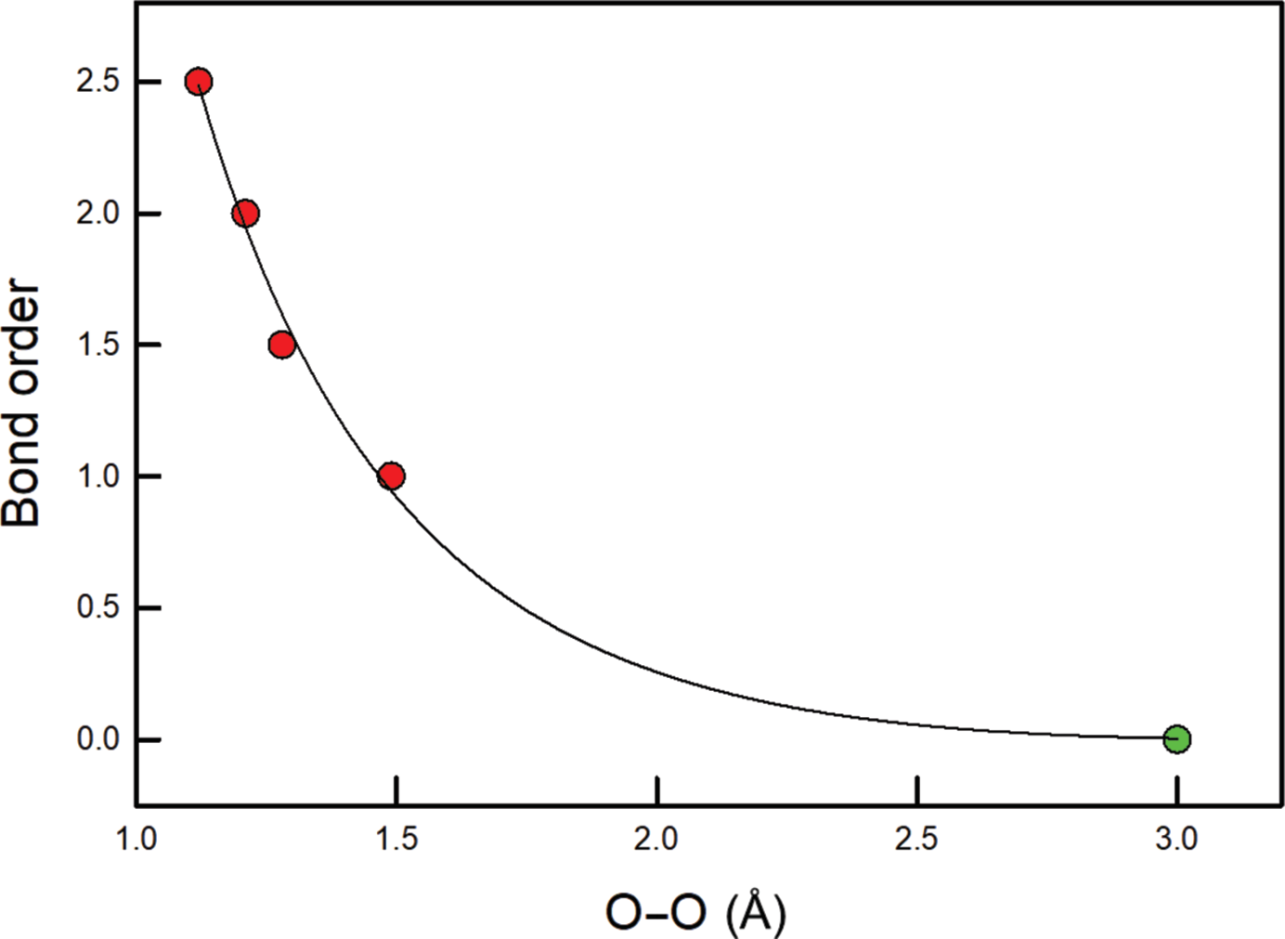
Variation in bond order as a function of O–O distance in the di­oxy­genyl cation (O_2_)^+^, di­oxy­gen (O_2_)^0^, superoxide (O_2_)^−^ and peroxide (O_2_)^2−^. The line is fit to the data and extrapolated to a distance of 3 Å for zero bond order; the value of 3 Å is somewhat speculative, but the resulting curve is not very sensitive to small changes in this value.

Fig. 9 ▸ shows the variation in bond order for the di­oxy­genyl cation (O_2_)^+^, di­oxy­gen (O_2_)^0^, superoxide (O_2_)^−^ and peroxide (O_2_)^2−^, versus O–O distance extrapolated to a distance of 3 Å for zero bond order. The O–O separations connected by bond paths are less than ∼3 Å and the extrapolated curve suggests bond orders of up to 0.15, and these numbers are not particularly sensitive to the distance assumed for zero bond order. The existence of attractive O–O interactions could possibly account for the unusual behaviour of bonded O radii for second-row cations in Fig. 7 ▸(*b*). In a footnote, Runtz *et al.* (1977 ▸, p. 3044) state the following: ‘It is possible that the sign of ∇^2^ρ(**r**) [where ρ(**r**) is the charge distribution] at the saddle point **r**, may be used to determine whether a given interaction is attractive or repulsive’. However, contrary to this view, Bader (1998 ▸, p. 7314) stated that ‘The presence of a bond path and its associated virial path provide a universal indicator of bonding between the atoms so linked. There is no net force acting on an element of ρ(**r**) or on an atom in a molecule in a stationary state, and ν(**r**) is attractive everywhere. Thus, contrary to what has appeared in the literature, no repulsive forces act on atoms linked by a bond path, nor on their nuclei.’

The basis of this approach, QTAIM (Quantum Theory of Atoms In a Molecule), is given in detail by Bader (1990 ▸) and QTAIM is widely used in the Chemistry community. However, it is by no means free of controversy (*e.g.* Poater *et al.*, 2006 ▸; Foroutan-Nejad *et al.*, 2014 ▸; Shahbazian, 2017 ▸; Jabłoński, 2019 ▸, 2023 ▸) and alternative interpretation. In view of the controversies and uncertainties surrounding QTAIM, its prediction of the behaviour of bonded-ion radii must still be considered uncertain, apart from the idea that current values of ionic radii and empirical ionic radii are not in accord with either quantum mechanical calculations or experimentally measured sizes of ions in crystals.

#### 4.2.2. Experimental bonded radii

Experimental electron-density distributions in crystal structures show ridges of electron density between nearest-neighbour atoms that are generally assumed to be chemically bonded together, and saddle points occur between nearest-neighbour atoms that are usually considered to be bonded together (*e.g.* Fig. 5 ▸). However, there has been no work that has derived ion radii from experimental electron densities covering all atoms of the Periodic Table. Experimental electron densities (*e.g.* Fig. 5 ▸) certainly suggest that atoms in crystals can be approximated by spheres. On the one hand, this contrasts with the space-filling shapes of the atomic basins into which the electron density is partitioned in QTAIM (Luaña *et al.*, 2003 ▸). As noted above, this issue has not been examined extensively by direct experimental measurement of electron-density distributions in crystals. For example, the presence or absence of a centre of symmetry at an atom position may drastically affect the polarization of the electron density of that atom, depending on the disposition of the atoms to which it is bonded. There are too many uncertainties with regard to any variation in the relative sizes of ions, a situation that can only be resolved by extensive measurement of electron-density distributions in crystals.

## 5. Mean interatomic distances and ion radii

Gagné & Hawthorne (2016 ▸, 2018*a* ▸,*b* ▸, 2020 ▸) and Gagné (2018 ▸, 2021 ▸) reported on the distribution of bond lengths to O^2−^ and N^3−^ in crystal structures refined since 1975 and listed in the Inorganic Crystal Structure Database (ICSD); O^2−^: for 135 ions bonded to oxygen in 459 configurations (on the basis of coordination number) using 177 143 bond lengths extracted from 30 805 ordered coordination polyhedra from 9210 crystal structures; N^3−^: for 76 ions bonded to nitro­gen in 137 configurations using 4048 bond lengths extracted from 875 ordered coordination polyhedra from 434 crystal structures. The O^2−^ data cover all ions of the Periodic Table and all observed coordination environments in which they occur in inorganic oxide and oxysalt compounds. The data were carefully filtered by hand to remove positional and chemical disorder, measurements done at non-ambient conditions, and obvious refinement errors. One result from this work is a set of grand mean bond lengths for all ions and coordination numbers; we will refer to these as *characteristic bond lengths*. Ion radii may be derived by subtracting a radius for O^2−^ from these characteristic bond lengths for cation-O^2−^ bonds.

### 5.1. Uses of ion/ionic radii

There are two broad types of use for ion radii: (1) those which compare the radii of cations with the radii of anions; (2) those which compare the radii of different cations or the radii of different anions. Methods belonging to type (1) use the relative sizes of cation and anion radii to predict local arrangements. As is apparent from the above discussion, derivation of the relative sizes (radii) of cations and anions cannot to date be done.

**Figure 10:**
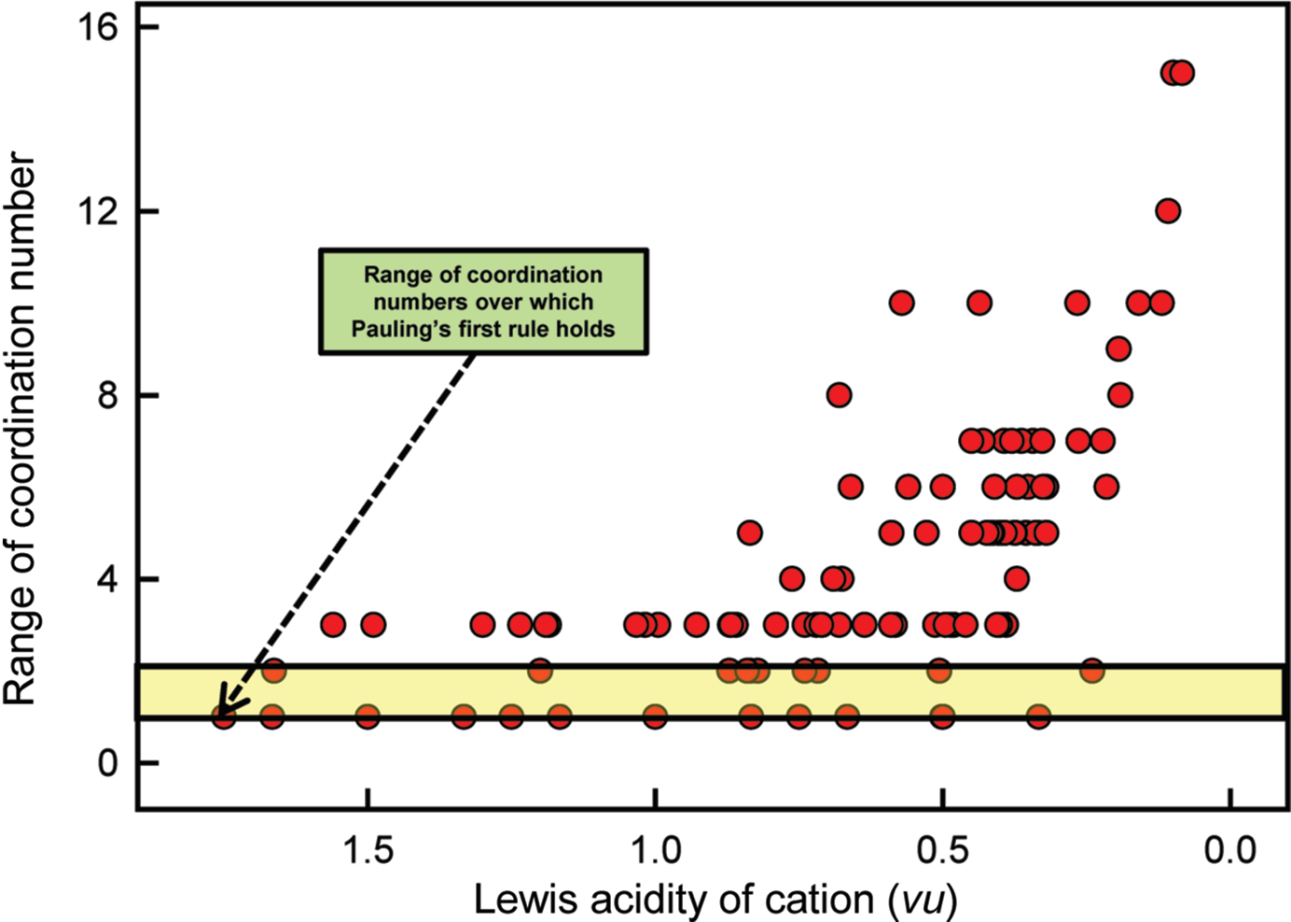
Variation in range of coordination number as a function of Lewis acidity for 135 cations; the yellow-shaded area denotes the maximum extent of data according to Pauling’s radius-ratio rule. Modified from Gibbs *et al.* (2022 ▸).

The classic type (1) method is the prediction of coordination number from the radius ratio of the constituent cation and anion. Hüttig (1920 ▸) proposed that the co­ordination number of a cation is determined by radius-ratio considerations and this became Pauling’s first rule (Pauling, 1929 ▸). For a single type of anion, Pauling’s first rule restricts the range of possible coordination numbers to 2: either the radius ratio is (i) close to a boundary value between two coordination numbers, in which case the cation can adopt either coordination number (*i.e.* there are two possible coordination numbers that it can have), or (ii) far away from a boundary value between two coordination numbers, in which case the cation has only one possible coordination number at ambient conditions. Fig. 10 ▸ examines the validity of this rule. It shows the range of coordination numbers adopted by different cations when bonded to O^2−^ as a function of the Lewis acidity (Gagné & Hawthorne, 2017*a* ▸) of the cation. Those cations that accord with the radius-ratio rule fall within the yellow region of Fig. 10 ▸ and are far outnumbered by the cations that do not accord with Pauling’s first rule, *i.e.* they have more than two observed coordination numbers. This lack of agreement shows either that the argument behind Pauling’s first rule is specious or that the cation and anion radii vary extensively with chemical composition and structure type; in either case, ion radii cannot be used in such a predictive manner.

**Figure 11:**
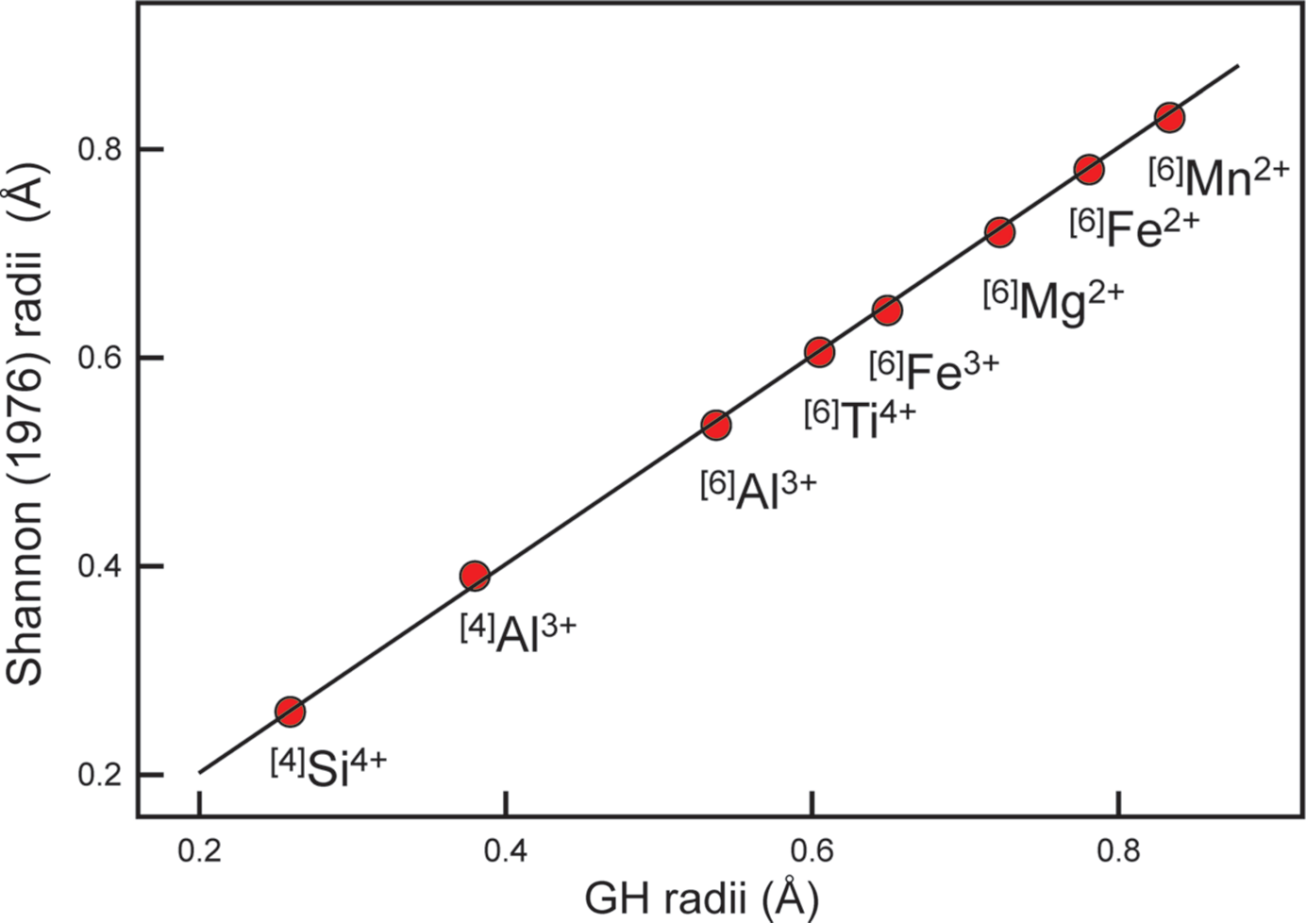
Comparison of the empirical ionic radii of Shannon (1976 ▸) with the ion radii derived here for the ion configurations ^[4]^Al^3+^, ^[4]^Si^4+^, ^[6]^Mg^2+^, ^[6]^Fe^2+^,^[6]^Mn^2+^, ^[6]^Al^3+^, ^[6]^Fe^3+^and ^[6]^Ti^4+^. The line denotes a 1:1 relation.

Methods belonging to type (2) use the relative sizes (radii) of cations and of anions but they do not rely on the radius ratio of cations and anions. Thus the fixed value for the radius of an anion used to derive the corresponding cation radii from observed interatomic distances does not affect the relative ordering of the cations with regard to their radii. For example, a common use of ion radii involves crystal structures in which there is extensive solid solution between two or more ions at a particular site in a structure. Relations between mean constituent ion radius and mean bond length for a particular site can be used to derive occupancies at that site for two ions with similar scattering factors, *e.g.* Si^4+^ and Al^3+^, and for more than two ions, *e.g.* Mg^2+^, Al^3+^ and Fe^3+^ where two ions have similar scattering factors and a third has a significantly different scattering factor. Such relations are linear with the mean bond lengths for ordered ion configurations and this linearity is not affected by the value of the O^2−^ radius used to derive the cation radii. So the issue here is what is the best value to use for the radius of O^2−^ to calculate ion radii. There are many equations developed to relate mean bond length to the aggregate ion radius of the constituents, and these equations are dependent on the actual value of the cation radii used. Most of these quantitative relations between mean bond length and mean empirical ionic radius involve a small number of ions in a small number of ion configurations: ^[4]^Al^3+^, ^[4]^Si^4+^, ^[6]^Mg^2+^, ^[6]^Fe^2+^, ^[6]^Mn^2+^, ^[6]^Al^3+^, ^[6]^Fe^3+^, ^[6]^Ti^4+^coordinated by O^2−^. It will be very advantageous if the new ion radii developed here for this small set of ion configurations have values close to those of Shannon (1976 ▸) as the numerous existing relations between mean bond length and mean empirical ionic radius can then still be used. Accordingly, we subtracted the Shannon radii for this small set of ions from their corresponding characteristic bond lengths (Table 2 ▸) to get the mean radius for O^2−^: 1.366 Å. The new radii are compared with the radii of Shannon (1976 ▸) for ^[4]^Al^3+^, ^[4]^Si^4+^, ^[6]^Mg^2+^, ^[6]^Fe^2+^, ^[6]^Mn^2+^,^[6]^Al^3+^, ^[6]^Fe^3+^, ^[6]^Ti^4+^ in Fig. 11 ▸ in which the values show a mean deviation of 0.003 Å. This radius for O^2−^, 1.366 Å, was subtracted from the characteristic bond length for each of the ion configurations in Table 2 ▸ to give the corresponding cation radii.

**Figure 12:**
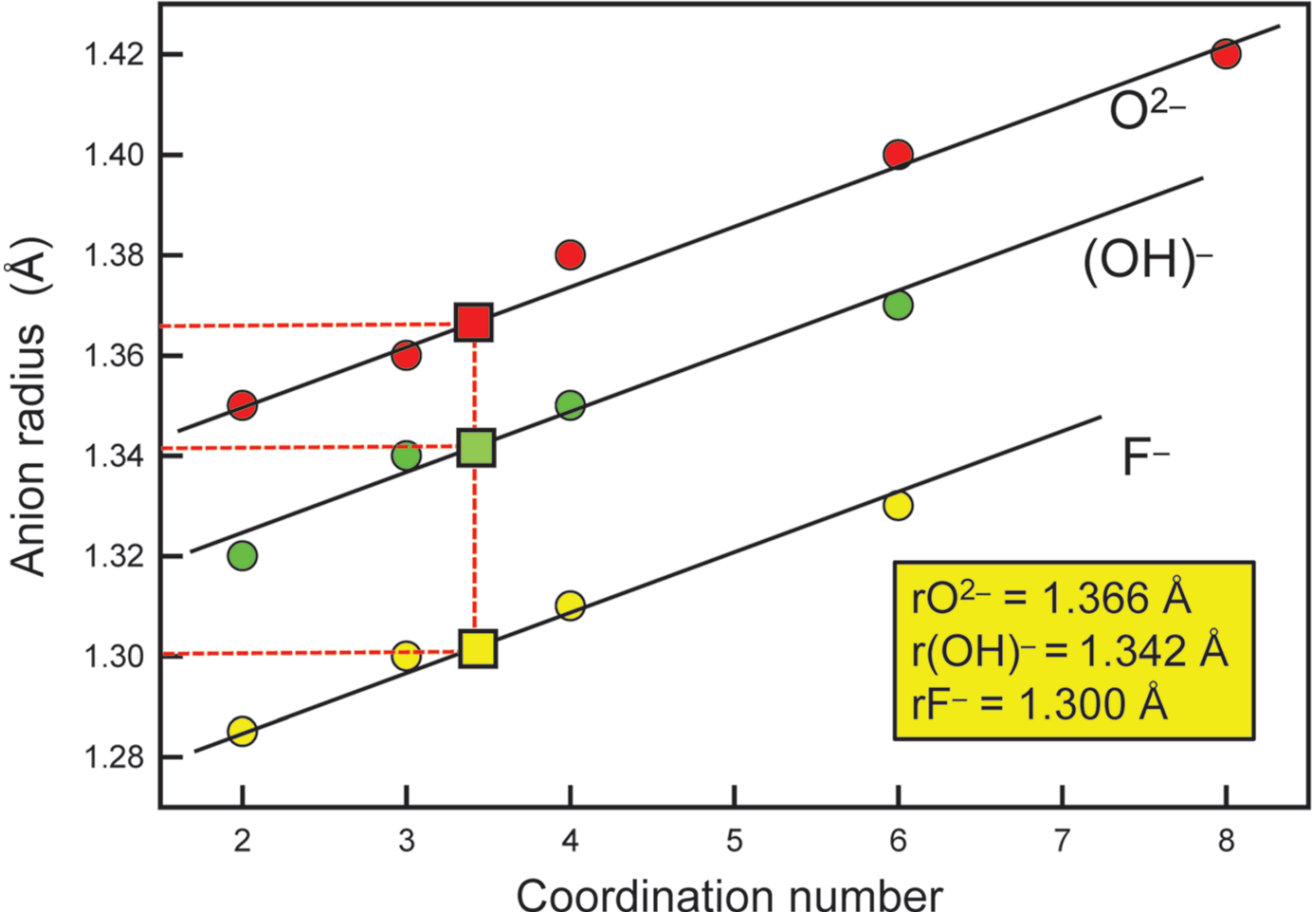
Variation in Shannon (1976 ▸) radii for O^2−^, (OH)^−^ and F^−^ as a function of anion coordination number. The red square denotes the value of the radius for O^2−^ derived here, and the green and yellow squares show the radii for (OH)^−^ and F^−^ that are consistent with the radius of 1.366 Å for O^2−^.

**Figure 13:**
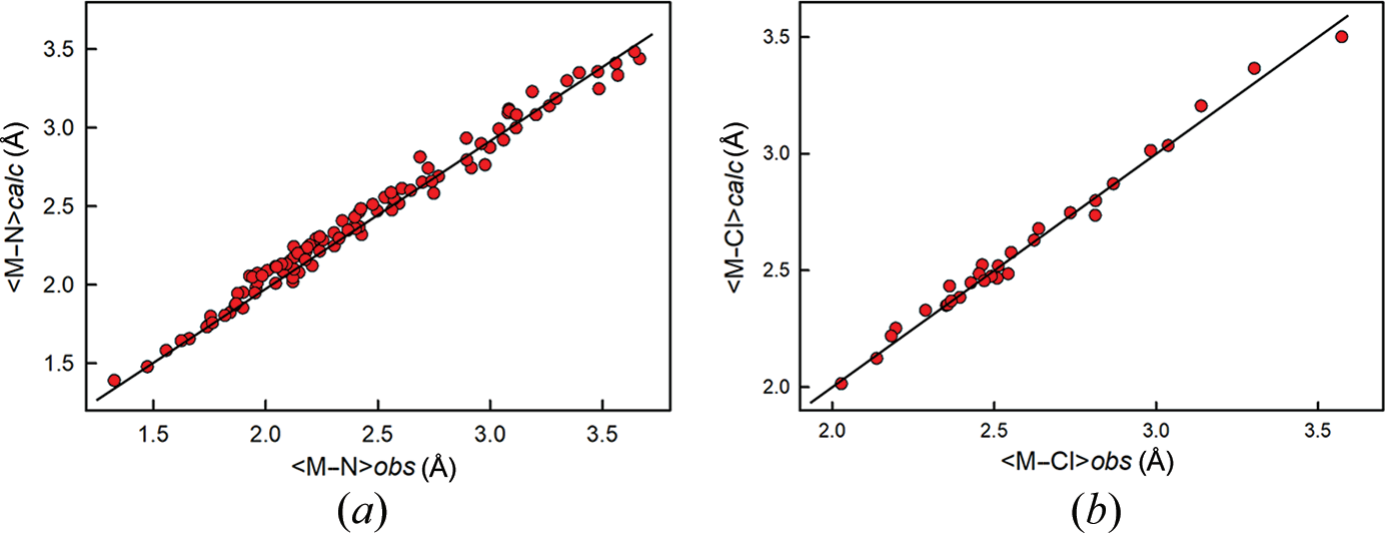
Comparison of observed mean bond lengths for the data used to derive the radii of N^3−^ and Cl^−^ with the sums of the ion radii for (*a*) 〈*M*–N〉 polyhedra and (*b*) 〈*M*–Cl〉 polyhedra.

The ion radii given in Table 2 ▸ can be made much more widely applicable by also having radii for other common anions, specifically N^3−^, (OH)^−^, F^−^ and Cl^−^. Shannon (1976 ▸) gives radii for (OH)^−^ and F^−^ in several coordinations, and these values are plotted as a function of coordination number in Fig. 12 ▸ in which we have fitted parallel lines to the trends and derived values for *r*(OH)^−^ and *r*F^−^ consistent with the value for *r*O^2−^ derived above: *r*(OH)^−^ = 1.342; *r*F^−^ = 1.300 Å. The radius for N^3−^ was derived as follows: Gagné (2021 ▸) lists grand 〈*M*–N^3−^〉 distances for 76 ions bonded to nitro­gen in 137 ion configurations. The grand 〈*M*–O^2−^〉 distances for the same 137 ion configurations was subtracted from the corresponding grand 〈*M*–N^3–^〉 distances and the mean of the resulting values is the difference between the ion radii of N^3−^ and O^2−^, giving *r*N^3–^ = 1.472 Å. The radius for Cl^−^ was derived in a similar fashion from a small set of interatomic distances in binary and ternary chlorides taken from ICSD: *r*Cl^−^ = 1.743 Å. The resulting ion radii are given in Table 2 ▸ and a comparison of the observed distances and sums of the constituent ion radii for the data used to derive the anion radii for N^3−^ and Cl^−^ are shown in Figs. 13 ▸(*a*) and 13 ▸(*b*).

## 6. Comparison of the current ion radii with previous values

**Figure 14:**
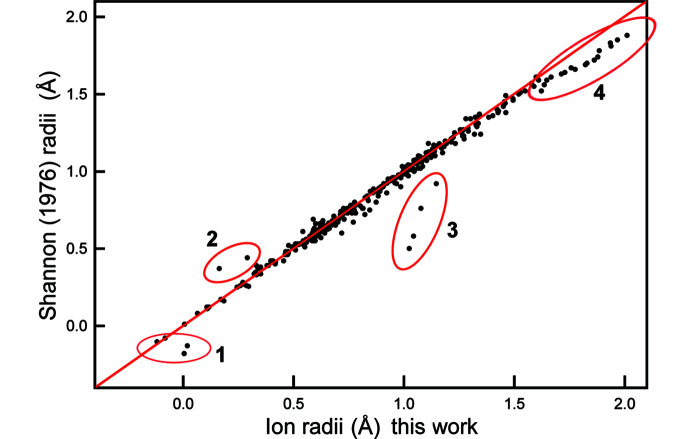
Variation in Shannon (1976 ▸) radii compared with the radii of Table 2 ▸. The red line denotes the 1:1 relation; significant deviations from the 1:1 relation are marked by red ellipses and these data are discussed in the text.

The current radii were derived entirely from experimental interatomic distances whereas previous compilations of ionic radii involved many values interpolated or extrapolated from correlations with other physical parameters. Table 2 ▸ contains ion radii for an additional 145 ion configurations relative to earlier compilations. The radii derived here are compared with the values of Shannon (1976 ▸) in Fig. 14 ▸. Most of the values lie along the 1:1 line, but there are notable deviations that are denoted by red ellipses in Fig. 14 ▸.

### 6.1. Ions possibly affected by O–O bonding

Consider first region 1 that corresponds to H^+^, ^[3]^C^4+^, ^[3]^N^5+^ and ^[4]^N^5+^. Gibbs *et al.* (2013 ▸, 2014 ▸) used Shannon’s negative radius of −0.18 Å for H^+^ as a criticism for using a fixed radius for O^2−^. However, the radius for H^+^ obtained here, 0.004 Å, is not negative. Both Shannon & Prewitt (1969 ▸) and Shannon (1976 ▸) used neutron diffraction data for deriving the radius for H^+^, but there was very little data available at that time and they had to use a combination of H–O and H–F distances, whereas Gagné & Hawthorne (2018*b* ▸) had 402 H—O bonds derived by neutron diffraction, and it seems reasonable to ascribe the difference in results to the relative availability of data. There are two negative radii in our list: −0.082 Å for ^[3]^C^4+^ and −0.119 Å for ^[3]^N^5+^. Inspection of Fig. 7 ▸ shows that the relation between the bonded radii for O and the bonded radii of the smaller second-row cations is very non-linear for C^4+^ and N^5+^, a feature that we suggest could be due to O–O bonding along the edges of the (CO_3_)^2−^ and (NO_3_)^1−^ oxy­anions as indicated by the occurrence of bond paths and bond critical points along those edges.

### 6.2. Radii affected by stereoactive lone-pair behaviour

Regions 2 (^[3]^P^3+^ and ^[3]^S^4+^) and 3 (^[6]^Se^4+^, ^[6]^As^3+^, ^[6]^Sb^3+^ and ^[8]^Li^+^) (Fig. 14 ▸) involve radii labelled A in Table 1 ▸ of Shannon (1976 ▸). These radii were not derived from interatomic distances directly by Shannon (1976 ▸) but were taken from Ahrens (1952 ▸) who calculated these ionic radii *via* extrapolation of relations between ionic radii and ionization potentials of the oxidation states of the corresponding ions. The differences between these and our radii for these ions are from 0.12 to 0.51 Å whereas the differences between these and our radii for other groups of ions is much less: for example, ^[6]^Ni^2+^,^[6]^Co^2+^, ^[6]^Fe^2+^ and ^[6]^Mn^2+^ differ by 0.014, 0.026, 0.036 and 0.033 Å. With the exception of ^[8]^Li^+^, the ions in regions 2 and 3 are lone-pair stereoactive. The large differences between the Ahrens radii and the radii of Table 2 ▸ for these ion configurations suggest that the relations used by Ahrens (1952 ▸) to derive ionic radii did not take into account (or are perturbed by) the presence of lone-pair stereoactivity and the formation of longer bonds on the side of the lone pair, as the resultant distances calculated from the sums of these radii and the empirical ionic radii of for O^2−^ given by Shannon (1976 ▸) do not accord with the corresponding characteristic distances of Table 2 ▸.

### 6.3. The origin of other deviations from linearity in Fig. 14 ▸

**Figure 15:**
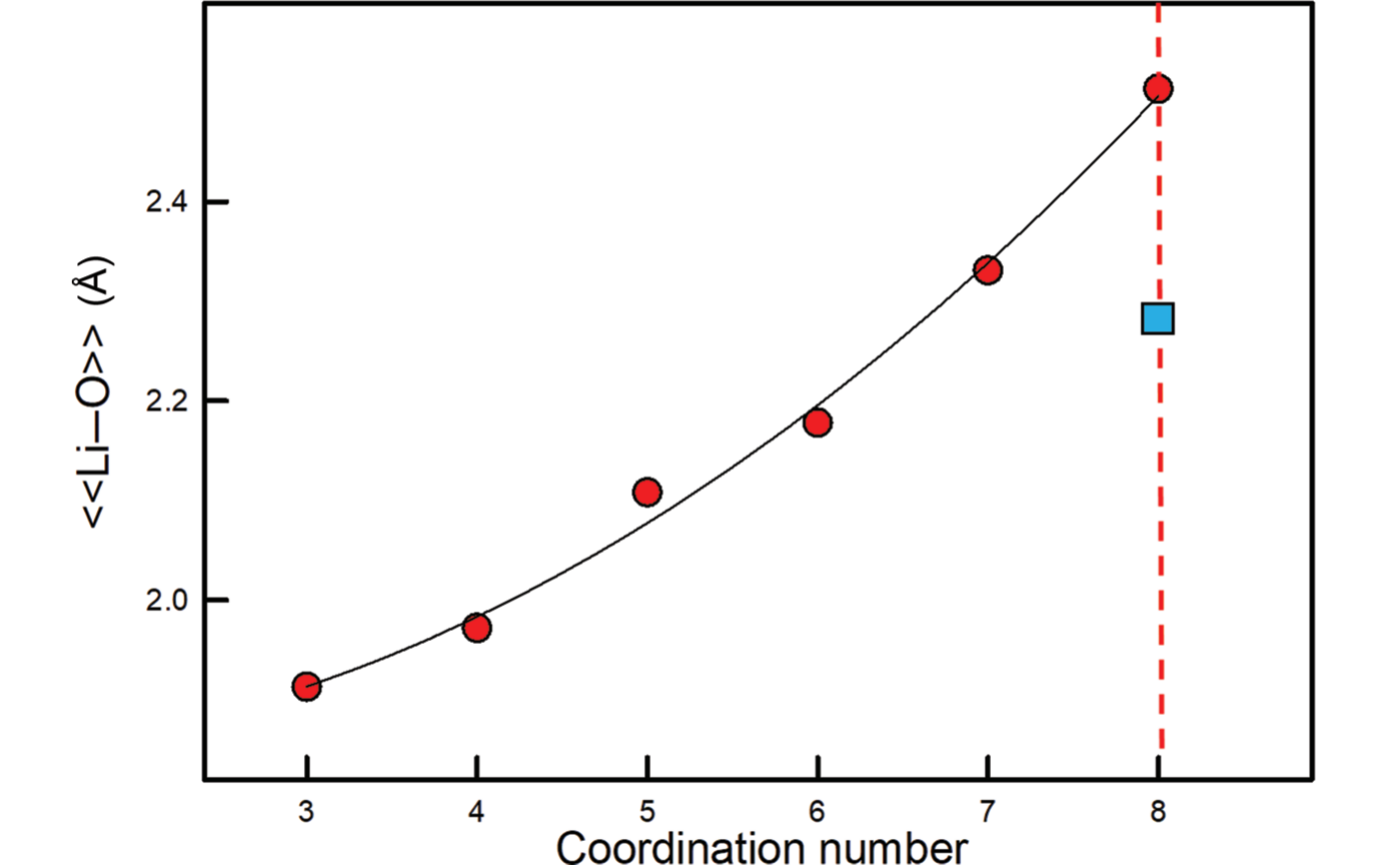
The variation in 〈〈Li–O〉〉 distance (red circles), values taken from Gagné & Hawthorne (2016 ▸), as a function of the coordination of Li. The blue square denotes the calculated radius of ^[8]^Li given by Shannon (1976 ▸).

Region 3 also contains ^[8]^Li+ which has significantly larger radius than that listed by Shannon (1976 ▸) which is a calculated value. Fig. 15 ▸ shows the variation in 〈〈Li^+^–O〉〉 (Table 2 ▸) as a function of coordination number of Li^+^. Our value for 〈〈^[8]^Li^+^–O〉〉 is based on one structure (Rb_6_LiPr_11_Cl_16_(SeO_3_)_12_, Lipp & Schleid, 2006 ▸) and fits a monotonic curve through the data for all observed coordination numbers for Li^+^ coordinated by O^2−^ whereas the previous value for 〈〈^[8]^Li^+^–O〉〉 deviates from this curve by ∼0.23 Å.

Region 4 contains K^+^, Rb^+^, Cs^+^ and Ba^2+^ with cation-coordination numbers higher than [12]. Gagné & Hawthorne (2015 ▸) gave bond-valence parameters for four ions to which they assigned coordination numbers higher than [12]: K^+^, Rb^+^, Cs^+^ and Ba^2+^. For comparison, Gagné & Hawthorne (2016 ▸) also derived new bond-valence parameters using a hard cut-off of 12 bonds for those configurations assigned coordination numbers greater than [12] by Gagné & Hawthorne (2015 ▸). Both sets of parameters (used in the way they were derived) gave exactly the same results for the anion bond-valence sums. Gagné & Hawthorne (2015 ▸) showed that (i) mean bond length is strongly correlated with *R*_o_ for all ions with multiple coordination numbers, and (ii) *R*_o_/(mean bond length) is correlated with ionization energy. They plotted mean bond length as a function of *R*_o_ for the five alkali-metal ions including and excluding bonds with a hard cut-off of [12]. Including the long bonds, *R*^2^ = 0.94, whereas excluding the long bonds, *R*^2^ drops to 0.79. Plotting *R*_o_/(mean bond length) against ionization energy, *R*^2^ (including long bonds) = 0.35, whereas *R*^2^ (excluding long bonds) = 0.01. From these results, Gagné & Hawthorne (2016 ▸) concluded that imposing a maximum coordination number of [12] on K^+^, Rb^+^, Cs^+^ and Ba^2+^ bonded to O^2−^ is not justified.

## 7. Ion radii as pr­oxy variables

### 7.1. Ion radii and Pauling’s first rule

**Figure 16:**
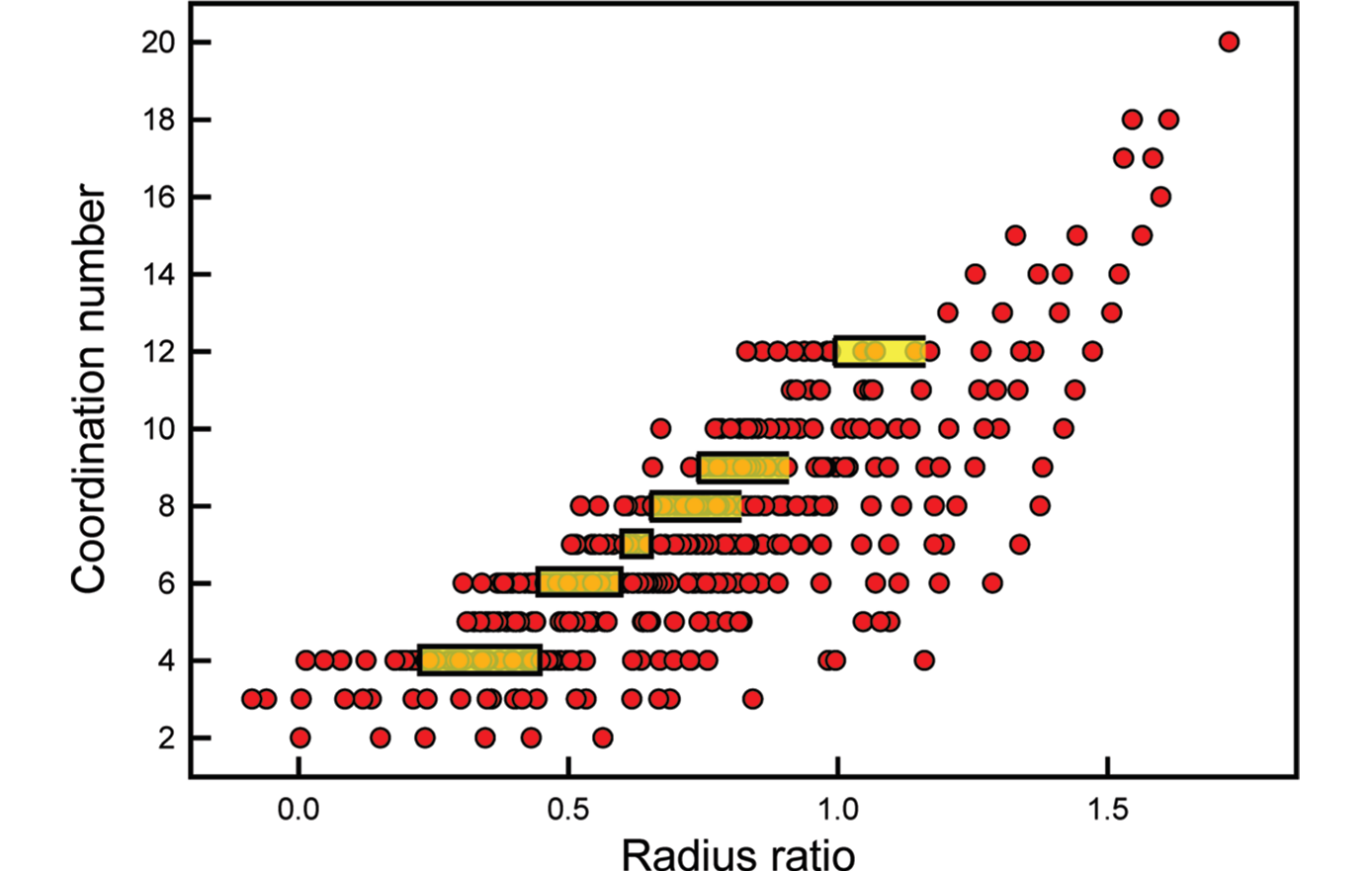
Variation in coordination number as a function of the radius ratio for 460 ion configurations; the yellow boxes denote the ranges in radius-ratio values given by Pauling (1960 ▸) for the corresponding coordination numbers.

It is apparent from the above discussion that ion radii, both cation and anion, are not fixed properties of ions. The assumption that they are fixed has led to much heated discussion in the literature, some of which has been discussed above. It is not necessary to appeal to detailed theoretical arguments or experimental electron-density data to show that arguments concerning the relative sizes of ions work where the predictions are approximate but fall apart on closer inspection. A very good example of this is Pauling’s first rule, the predictive capabilities of which are poor as discussed in Section 5. If we wish to explain the observed coordination numbers for specific ions, we should use the mean radii for each separate ion and coordination number. This is done in Fig. 16 ▸ for the radii of Table 2 ▸; the limiting radius ratio for each coordination listed by Pauling (1960 ▸) is marked by the yellow boxes. It is apparent that Pauling’s first rule fails to explain the observed coordination numbers even where the radii used are specific to those coordination numbers. Fig. 16 ▸ indicates that we cannot consider ions as hard spheres of fixed radii that behave according to the geometrical content of Pauling’s first rule.

As discussed above, bonded radii are much more variable (Figs. 6 ▸ and 7 ▸) and we must also consider these radii in the context of Pauling’s first rule. As is evident from Fig. 6 ▸, both cations and anions show considerable variation in radii with coordination. Thus Mg^2+^ coordinated by O^2−^ shows the following ranges in bonded radii: 0.72 < Mg^2+^ < 0.78 Å; 1.07 < O^2−^ < 1.36 Å, initially suggesting much more variation in radius ratio than is the case for fixed radii. Such variation could possibly give rise to the large range of coordination numbers apparent in Fig. 11 ▸. However, the variations in cation and anion radii in Fig. 6 ▸ show perfect positive correlations and the radius ratio for Mg^2+^ bonded to O^2−^ shows a change of only 0.06, a very small value that is incompatible with the ranges indicated in Fig. 16 ▸.

### 7.2. Ion radii as pr­oxy variables for mean bond lengths

The idea of ion radii is physically appealing because of its ostensible simplicity, and ion radii played a major role in early structural crystallography by aiding in the solution of crystal structures and by helping to systematize our knowledge of crystal structure arrangements. However, it is apparent from the discussions above that the rationale underlying these uses is approximate at best, despite the persuasive appearance of electron density distributions in crystals (*e.g*. Fig. 5 ▸) and the compelling theoretical basis for bond critical points and bonded radii.

Certain applications of ion radii are extremely precise and produce results that are very accurate whereas other uses are at best semi-quantitative. We may recognize two distinct categories of usage. Type (1), using ratios of cation radii to anion radii to predict or correlate ion arrangements and/or physical properties, *e.g*. coordination number; such relations are not very accurate, *e.g*. Fig. 10 ▸. Type (2), using cation-radii (or anion-radii) or sums of cation and anion radii to predict or correlate ion arrangements and/or physical properties, *e.g*. observed mean bond lengths, site populations in crystals; such relations can be very precise and are essential for producing site populations in crystals of complicated chemical composition, particularly where ion constituents have very similar scattering factors.

We may write the relation between mean interatomic distance and the radii of the constituent ions, *A*^+^ and *B*^−^, as follows: 〈*A*–*B*〉 = *rA*^+^ + *rB*^−^ where 〈*A*–*B*〉 is the mean interatomic distance between the constituent ions, and *rA*^+^ and *rB*^−^ are the corresponding ion radii. Generally we know 〈*A*–*B*〉 very accurately, and the problem involves deriving accurate values for *rA*^+^ and *rB*^−^. If we try to develop relations involving the ratio of cation radii to anion radii, these relations will not be accurate, whereas if we develop relations involving sums of cation radii and/or sums of anion radii, we do not need to know accurate values of the ion radii because the relation 〈*A*–*B*〉 = *rA*^+^ + *rB*^−^ (or its more complicated analogues) means that *rA*^+^ (or *rA*^+^ and *rB*^−^) is a *pr­oxy variable* for 〈*A*–*B*〉, the corresponding mean bond lengths associated with *rA*^+^ and *rB*^−^ (Table 2 ▸).

**Figure 17:**
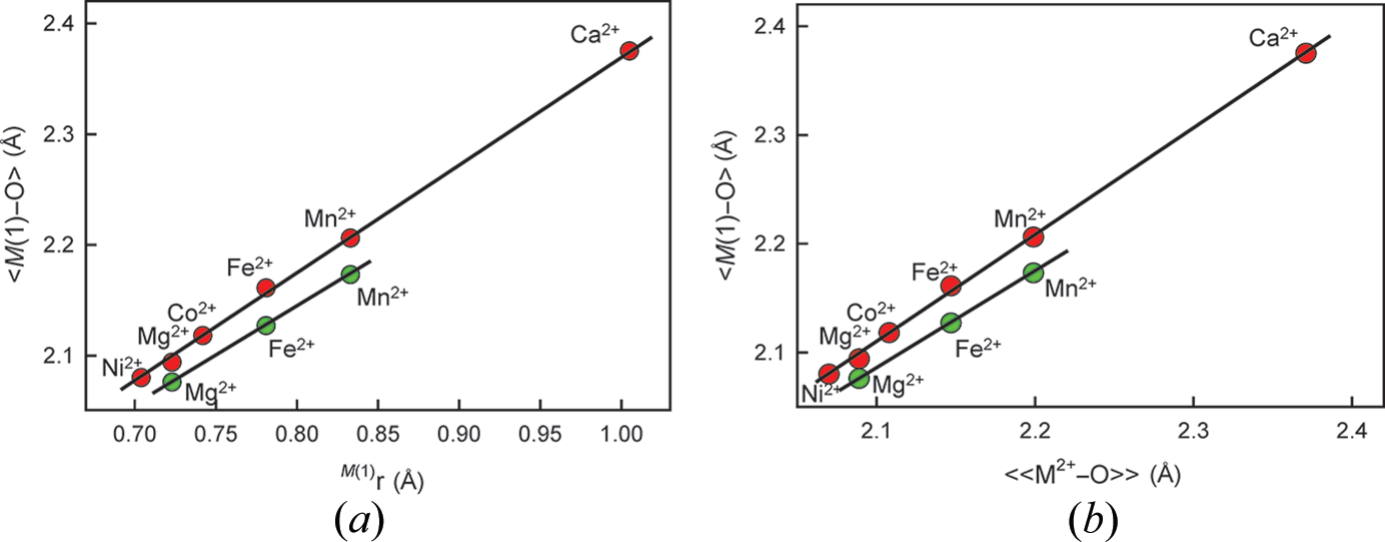
〈*M*(1)–O〉 in olivines (red circles): *M*^2+^_2_SiO_4_, where *M*(1) = Ni, Mg, Co, Fe, Mn, Ca; and Ca-dominant clinopyroxenes (green circles): Ca*M*^2+^Si_2_O_6_, where *M*(1) = Mg, Fe, Mn; (*a*) 〈*M*(1)–O〉 *versus ^M^*^(1)^*r*; (*b*) 〈*M*(1)–O〉 *versus* 〈〈^[6]^*M*^2+^–O^2−^〉〉 (characteristic distances for inorganic structures).

To illustrate this point, consider Fig. 17 ▸. Fig. 17 ▸(*a*) shows the variation in 〈*M*(1)–O〉 distance for ordered olivine structures *M*^2+^_2_SiO_4_ as a function of *^M^*^(1)^*r* where *M*^2+^ = Ni^2+^, Mg^2+^, Co^2+^, Fe^2+^, Mn^2+^ and Ca^2+^, and Fig. 17 ▸(*b*) shows the variation in 〈*M*(1)–O〉 distance for the same set of ordered olivine structures as a function of characteristic 〈^[6]^*M*^2+^–O〉 distance from Table 2 ▸ for the same set of ions; both relations are linear. In Fig. 17 ▸(*a*), we have set *r*O^2−^ to 1.366 Å; in Fig. 17 ▸(*b*), use of the characteristic bond length implies that we have set *r*^cation^ to the corresponding characteristic bond length and *r*O^2−^ to 0.000 Å. Whatever value of anion radius we choose, the corresponding set of cation radii are just a set of pr­oxy variables for the corresponding characteristic bond lengths.

Gagné & Hawthorne (2017*b* ▸, 2020 ▸) showed that variation in bond-topological asymmetry is the most important factor affecting the variation of mean bond lengths of a particular ion configuration in crystal structures. For a specific structure type, *e.g*. olivine, there is no difference in bond-topological asymmetry and hence bond lengths are affected only by the sizes and any intrinsic electronic effects of the constituent ions. If we choose a different structure type with a different bond-topological asymmetry, *e.g*. pyroxene, again bond lengths are affected only by the sizes and any intrinsic electronic effects of the constituent ions. However, the linear relations between the two structure types will be different because of the difference in bond-topological asymmetry. This is illustrated in Fig. 17 ▸: there are precise linear relations for the olivine (red circles) and pyroxene (green circles) structures, but the positions and slopes of the two lines are subtly different, reflecting the difference in bond-topological asymmetry of the two structure types.

## 8. Coda

[1] The ion radii listed here were derived entirely from experimentally determined (and filtered) interatomic distances.

[2] There are radii for an additional 145 explicitly determined ion configurations compared to previous listings of empirical ionic radii.

[3] Ion radii have significant restraints on their use:

(i) They are not effective for applications of type (1) involving ratios of cation and anion radii (*e.g.* prediction of coordination number).

(ii) Radii are far more effective for type (2) applications that involve the relative radii of cations or of anions (*e.g.* prediction of mean bond lengths, derivation of site occupancies).

(iii) Applications of type (2) that involve sums of radii can be extremely accurate when dealing with isotypic structures (*i.e*. those that have identical long-range bond topology).

(iv) Applications of type (2) are less accurate when dealing with non-isotypic structures (*i.e*. those that do not have identical long-range bond topology), as variation in bond-topological asymmetry is a major factor affecting the variation of mean bond lengths in crystal structures (Gagné & Hawthorne, 2020 ▸).

(v) As a result of (iv), users must be aware of the considerable variation that mean bond lengths (and thus ion radii) exhibit across structure type, and the limitations that this may impose on the accuracy of any trends and models based on ion radii.

[4] Ion radii are pr­oxy variables for characteristic bond lengths. They are not effective in type (1) applications but are very effective in type (2) applications.

## Figures and Tables

**Table 1:** Charges at atoms in *M*^2+^_2_Si_2_O_6_ pyroxene structures determined by charge-density refinement of X-ray diffraction data†

	Mg^2+^	Fe^2+^	Co^2+^
*M* ^2+^	+1.82	+1.12	+0.95
Si^4+^	+2.28	+2.28	+2.19
O^2−^_term_	−1.42	−1.13	−1.13
O^2−^_br_	−1.27	−0.97	−1.05
O^2−^	−1.37	−1.10	−1.08

† Values from Sasaki *et al.* (1982 ▸).

**Table 2:** Characteristic bond lengths and ion radii derived from experimental interatomic distances [CN = coordination number]

*Z*	Ion	CN	〈〈Bond length〉〉 (Å)	Ion radius (Å)	*Z*	Ion	CN	〈〈Bond length〉〉 (Å)	Ion radius (Å)	*Z*	Ion	CN	〈〈Bond length〉〉 (Å)	Ion radius (Å)	*Z*	Ion	CN	〈〈Bond length〉〉 (Å)	Ion radius (Å)
1	H^+^	[2]	1.370	0.004	22	Ti^3+^	[6]	2.037	0.671	31	Ga^3+^	[4]	1.842	0.476	42	Mo^3+^	[6]	2.095	0.729
		[3]	1.916	0.550			[7]	2.108	0.742			[5]	1.910	0.544		Mo^4+^	[6]	2.003	0.637
		[4]	2.232	0.866			[8]	2.195	0.829			[6]	1.979	0.613		Mo^5+^	[5]	1.916	0.550
3	Li^+^	[3]	1.913	0.547		Ti^4+^	[4]	1.821	0.455	32	Ge^4+^	[4]	1.752	0.386			[6]	1.992	0.626
		[4]	1.972	0.606			[5]	1.917	0.551			[5]	1.847	0.481		Mo^6+^	[4]	1.764	0.398
		[5]	2.108	0.742			[6]	1.971	0.605			[6]	1.895	0.529			[5]	1.872	0.506
		[6]	2.178	0.812			[7]	2.064	0.698	33	As^3+^	[3]	1.776	0.410			[6]	1.973	0.607
		[7]	2.331	0.965	23	V^3+^	[6]	2.007	0.641			[4]	2.026	0.660	43	Tc^7+^	[4]	1.705	0.339
		[8]	2.513	1.147		V^4+^	[5]	1.893	0.527			[5]	2.247	0.881	44	Ru^3+^	[6]	2.025	0.659
4	Be^2+^	[3]	1.550	0.184			[6]	1.980	0.614			[6]	2.410	1.044		Ru^4+^	[6]	1.982	0.616
		[4]	1.638	0.272		V^5+^	[4]	1.717	0.351			[8]	2.480	1.114		Ru^5+^	[6]	1.964	0.598
5	B^3+^	[3]	1.372	0.006			[5]	1.827	0.461		As^5+^	[4]	1.688	0.322	45	Rh^3+^	[6]	2.025	0.659
		[4]	1.475	0.109			[6]	1.924	0.558			[6]	1.830	0.464		Rh^4+^	[6]	2.007	0.641
6	C^4+^	[3]	1.285	−0.082	24	Cr^2+^	[4]	2.004	0.638	34	Se^4+^	[3]	1.691	0.325	46	Pd^2+^	[4]	2.011	0.645
7	N^5+^	[3]	1.247	−0.119			[5]	2.113	0.747			[4]	2.027	0.661		Pd^4+^	[6]	1.999	0.633
		[4]	1.385	0.019			[6]	2.188	0.822			[5]	2.237	0.871	47	Ag^+^	[2]	2.136	0.770
11	Na^+^	[3]	2.307	0.941		Cr^3+^	[6]	1.976	0.610			[6]	2.390	1.024			[3]	2.278	0.912
		[4]	2.359	0.993		Cr^4+^	[4]	1.784	0.418			[7]	2.503	1.137			[4]	2.402	1.036
		[5]	2.413	1.047			[6]	1.950	0.584			[8]	2.530	1.164			[5]	2.489	1.123
		[6]	2.441	1.075		Cr^5+^	[4]	1.693	0.327			[9]	2.728	1.362			[6]	2.537	1.171
		[7]	2.541	1.175		Cr^6+^	[4]	1.652	0.286			[10]	2.882	1.516			[7]	2.589	1.223
		[8]	2.599	1.233	25	Mn^2+^	[4]	2.046	0.680		Se^6+^	[4]	1.636	0.270			[8]	2.656	1.290
		[9]	2.686	1.320			[5]	2.141	0.775	35	Br^5+^	[6]	2.281	0.915			[9]	2.704	1.338
		[10]	2.741	1.375			[6]	2.199	0.833			[7]	2.578	1.212	48	Cd^2+^	[5]	2.257	0.891
		[12]	2.795	1.429			[7]	2.352	0.986			[8]	2.671	1.305			[6]	2.302	0.936
12	Mg^2+^	[4]	1.939	0.573			[8]	2.321	0.955		Br^7+^	[4]	1.611	0.245			[7]	2.377	1.011
		[5]	1.966	0.600		Mn^3+^	[4]	1.901	0.535	37	Rb^+^	[4]	2.951	1.585			[8]	2.432	1.066
		[6]	2.089	0.723			[5]	1.959	0.593			[5]	2.864	1.498			[9]	2.530	1.164
		[8]	2.255	0.889			[6]	2.031	0.665			[6]	2.989	1.623	49	In^3+^	[6]	2.142	0.776
13	Al^3+^	[4]	1.746	0.380		Mn^4+^	[4]	1.750	0.384			[7]	3.002	1.636			[7]	2.218	0.852
		[5]	1.842	0.476			[6]	1.903	0.537			[8]	3.033	1.667			[8]	2.275	0.909
		[6]	1.903	0.537		Mn^5+^	[4]	1.698	0.332			[9]	3.079	1.713	50	Sn^2+^	[3]	2.094	0.728
14	Si^4+^	[4]	1.625	0.259		Mn^6+^	[4]	1.662	0.296			[10]	3.142	1.776			[4]	2.281	0.915
		[6]	1.783	0.417		Mn^7+^	[4]	1.610	0.244			[11]	3.188	1.822			[5]	2.451	1.085
15	P^3+^	[3]	1.656	0.290	26	Fe^2+^	[3]	1.844	0.478			[12]	3.228	1.862			[6]	2.508	1.142
	P^5+^	[4]	1.537	0.171			[4]	1.985	0.619			[13]	3.293	1.927			[7]	2.690	1.324
16	S^4+^	[3]	1.529	0.163			[5]	2.097	0.731			[14]	3.301	1.935			[8]	2.706	1.340
S^6+^		[4]	1.473	0.107			[6]	2.147	0.781			[15]	3.338	1.972			[9]	2.828	1.462
17	Cl^3+^	[2]	1.573	0.207			[8]	2.333	0.967			[17]	3.456	2.090		Sn^4+^	[4]	1.956	0.590
		[4]	2.233	0.867		Fe^3+^	[4]	1.875	0.509			[18]	3.478	2.112			[6]	2.054	0.688
	Cl^5+^	[3]	1.483	0.117			[5]	1.966	0.600	38	Sr^2+^	[6]	2.477	1.111			[7]	2.115	0.749
	Cl^7+^	[4]	1.431	0.065			[6]	2.015	0.649			[7]	2.639	1.273	51	Sb^3+^	[3]	1.932	0.566
19	K^+^	[4]	2.708	1.342			[8]	2.125	0.759			[8]	2.658	1.292			[4]	2.092	0.726
		[5]	2.796	1.430	27	Co^2+^	[3]	1.854	0.488			[9]	2.703	1.337			[5]	2.240	0.874
		[6]	2.828	1.462			[4]	1.967	0.601			[10]	2.769	1.403			[6]	2.443	1.077
		[7]	2.861	1.495			[5]	2.066	0.700			[11]	2.798	1.432			[7]	2.486	1.120
		[8]	2.894	1.528			[6]	2.108	0.742			[12]	2.825	1.459			[8]	2.584	1.218
		[9]	2.956	1.590			[8]	2.272	0.906	39	Y^3+^	[6]	2.264	0.898			[9]	2.758	1.392
		[10]	3.013	1.647		Co^3+^	[6]	1.908	0.542			[7]	2.332	0.966		Sb^5+^	[6]	1.977	0.611
		[11]	3.089	1.723		Co^4+^	[6]	1.874	0.508			[8]	2.390	1.024	52	Te^4+^	[3]	1.843	0.477
		[12]	3.095	1.729	28	Ni^2+^	[2]	1.686	0.320			[9]	2.422	1.056			[4]	1.984	0.618
		[13]	3.149	1.783			[4]	1.950	0.584			[10]	2.496	1.130			[5]	2.251	0.885
		[14]	3.239	1.873			[5]	2.028	0.662			[12]	2.541	1.175			[6]	2.386	1.020
		[15]	3.182	1.816			[6]	2.070	0.704	40	Zr^4+^	[6]	2.078	0.712			[7]	2.460	1.094
20	Ca^2+^	[6]	2.371	1.005		Ni^4+^	[6]	1.870	0.504			[7]	2.146	0.780			[8]	2.594	1.228
		[7]	2.447	1.081	29	Cu^+^	[2]	1.839	0.473			[8]	2.200	0.834			[9]	2.677	1.311
		[8]	2.498	1.132			[3]	1.969	0.603			[9]	2.263	0.897			[10]	2.833	1.467
		[9]	2.559	1.193			[4]	2.084	0.718			[10]	2.283	0.917			[11]	2.812	1.446
		[10]	2.632	1.266		Cu^2+^	[4]	1.943	0.577	41	Nb^4+^	[6]	2.054	0.688			[12]	2.928	1.562
		[11]	2.614	1.248			[5]	2.037	0.671		Nb^5+^	[4]	1.831	0.465		Te^6+^	[6]	1.923	0.557
		[12]	2.668	1.302			[6]	2.130	0.764			[5]	1.926	0.560	53	I^5+^	[6]	2.294	0.928
21	Sc^3+^	[6]	2.098	0.732			[8]	2.302	0.936			[6]	1.993	0.627			[7]	2.438	1.072
		[7]	2.163	0.797		Cu^3+^	[4]	1.846	0.480			[7]	2.069	0.703			[8]	2.587	1.221
		[8]	2.234	0.868	30	Zn^2+^	[4]	1.952	0.586			[8]	2.080	0.714			[9]	2.699	1.333
							[5]	2.051	0.685							I^7+^	[4]	1.763	0.397
							[6]	2.110	0.744								[6]	1.892	0.526
55	Cs^+^	[6]	3.124	1.758	62	Sm^3+^	[6]	2.352	0.986	72	Hf^4+^	[6]	2.082	0.716	82	Pb^2+^	[3]	2.210	0.844
		[7]	3.193	1.827			[7]	2.406	1.040			[7]	2.128	0.762			[4]	2.357	0.991
		[8]	3.244	1.878			[8]	2.450	1.084			[8]	2.190	0.824			[5]	2.482	1.116
		[9]	3.251	1.885			[9]	2.494	1.128	73	Ta^5+^	[6]	1.988	0.622			[6]	2.581	1.215
		[10]	3.304	1.938			[10]	2.560	1.194			[7]	2.057	0.691			[7]	2.637	1.271
		[11]	3.333	1.967			[12]	2.714	1.348	74	W^5+^	[6]	1.956	0.590			[8]	2.697	1.331
		[12]	3.377	2.011	63	Eu^2+^	[8]	2.628	1.262		W^6+^	[4]	1.773	0.407			[9]	2.750	1.384
		[13]	3.426	2.060			[9]	2.693	1.327			[5]	1.859	0.493			[10]	2.789	1.423
		[14]	3.444	2.078		Eu^3+^	[7]	2.391	1.025			[6]	1.951	0.585			[11]	2.821	1.455
		[15]	3.503	2.137			[8]	2.432	1.066	75	Re^5+^	[6]	1.940	0.574			[12]	2.828	1.462
		[16]	3.550	2.184			[9]	2.467	1.101		Re^7+^	[4]	1.716	0.350		Pb^4+^	[4]	2.056	0.690
		[17]	3.530	2.164			[10]	2.530	1.164			[5]	1.810	0.444			[5]	2.147	0.781
		[18]	3.570	2.204	64	Gd^3+^	[6]	2.304	0.938			[6]	1.883	0.517			[6]	2.169	0.803
		[20]	3.723	2.357			[7]	2.375	1.009	76	Os^5+^	[6]	1.960	0.594	83	Bi^3+^	[3]	2.069	0.703
56	Ba^2+^	[6]	2.689	1.323			[8]	2.422	1.056		Os^6+^	[6]	1.926	0.560			[4]	2.212	0.846
		[7]	2.793	1.427			[9]	2.476	1.110		Os^7+^	[5]	1.825	0.459			[5]	2.317	0.951
		[8]	2.816	1.450			[10]	2.514	1.148			[6]	1.887	0.521			[6]	2.398	1.032
		[9]	2.860	1.494			[11]	2.627	1.261		Os^8+^	[4]	1.698	0.332			[7]	2.497	1.131
		[10]	2.915	1.549	65	Tb^3+^	[6]	2.289	0.923			[5]	1.793	0.427			[8]	2.522	1.156
		[11]	2.944	1.578			[7]	2.358	0.992			[6]	1.885	0.519			[9]	2.604	1.238
		[12]	2.965	1.599			[8]	2.400	1.034	77	Ir^3+^	[6]	2.042	0.676			[10]	2.670	1.304
		[13]	3.010	1.644			[9]	2.440	1.074		Ir^4+^	[4]	1.909	0.543			[12]	2.671	1.305
		[14]	3.080	1.714			[10]	2.513	1.147			[6]	2.015	0.649		Bi^5+^	[4]	1.979	0.613
57	La^3+^	[6]	2.451	1.085		Tb^4+^	[6]	2.181	0.815		Ir^5+^	[6]	1.990	0.624			[6]	2.110	0.744
		[7]	2.507	1.141	66	Dy^3+^	[6]	2.275	0.909	78	Pt^2+^	[4]	2.007	0.641	90	Th^4+^	[8]	2.425	1.059
		[8]	2.548	1.182			[7]	2.353	0.987		Pt^4+^	[6]	2.021	0.655			[9]	2.462	1.096
		[9]	2.586	1.220			[8]	2.392	1.026	79	Au^3+^	[4]	1.999	0.633			[10]	2.505	1.139
		[10]	2.635	1.269			[9]	2.446	1.080	80	Hg^2+^	[2]	1.955	0.589			[12]	2.580	1.214
		[11]	2.662	1.296			[10]	2.482	1.116			[4]	2.316	0.950	92	U^4+^	[7]	2.318	0.952
		[12]	2.705	1.339	67	Ho^3+^	[6]	2.263	0.897			[5]	2.380	1.014			[8]	2.379	1.013
58	Ce^3+^	[7]	2.469	1.103			[7]	2.336	0.970			[6]	2.429	1.063			[9]	2.427	1.061
		[8]	2.495	1.129			[8]	2.387	1.021			[7]	2.505	1.139			[10]	2.460	1.094
		[9]	2.553	1.187			[9]	2.411	1.045			[8]	2.502	1.136			[12]	2.501	1.135
		[10]	2.595	1.229			[10]	2.499	1.133	81	Tl^+^	[3]	2.517	1.151		U^5+^	[6]	2.131	0.765
		[11]	2.686	1.320	68	Er^3+^	[6]	2.252	0.886			[4]	2.726	1.360			[7]	2.250	0.884
		[12]	2.647	1.281			[7]	2.321	0.955			[5]	2.840	1.474		U^6+^	[6]	2.110	0.744
	Ce^4+^	[6]	2.214	0.848			[8]	2.364	0.998			[6]	2.887	1.521			[7]	2.205	0.839
		[8]	2.345	0.979			[9]	2.414	1.048			[7]	2.976	1.610			[8]	2.288	0.922
		[9]	2.393	1.027			[10]	2.436	1.070			[8]	2.977	1.611	93	Np^5+^	[6]	2.211	0.845
		[10]	2.436	1.070	69	Tm^3+^	[6]	2.250	0.884			[9]	2.991	1.625			[7]	2.283	0.917
		[12]	2.502	1.136			[7]	2.317	0.951			[10]	3.102	1.736			[8]	2.369	1.003
59	Pr^3+^	[7]	2.441	1.075			[8]	2.362	0.996			[11]	3.134	1.768		Np^6+^	[7]	2.189	0.823
		[8]	2.478	1.112			[9]	2.418	1.052			[12]	3.195	1.829			[8]	2.261	0.895
		[9]	2.526	1.160			[10]	2.430	1.064		Tl^3+^	[6]	2.228	0.862		Np^7+^	[6]	2.048	0.682
		[10]	2.613	1.247	70	Yb^3+^	[6]	2.242	0.876		Tl^3+^	[7]	2.336	0.970	95	Am^3+^	[9]	2.503	1.137
		[11]	2.688	1.322			[7]	2.325	0.959			[8]	2.378	1.012	96	Cm^3+^	[9]	2.490	1.124
		[12]	2.672	1.306			[8]	2.357	0.991										
60	Nd^3+^	[6]	2.365	0.999			[9]	2.385	1.019						8	O^2−^			1.366
		[7]	2.447	1.081			[10]	2.421	1.055							(OH)^−^			1.342
		[8]	2.478	1.112	71	Lu^3+^	[6]	2.226	0.860						7	F^−^			1.300
		[9]	2.512	1.146			[7]	2.295	0.929						17	Cl^–^			1.743
		[10]	2.585	1.219			[8]	2.342	0.976						7	N^3−^			1.472
		[12]	2.622	1.256			[9]	2.359	0.993										
							[10]	2.516	1.150										
